# A systematic structural study of halogen bonding *versus* hydrogen bonding within competitive supramolecular systems

**DOI:** 10.1107/S2052252515010854

**Published:** 2015-07-30

**Authors:** Christer B. Aakeröy, Christine L. Spartz, Sean Dembowski, Savannah Dwyre, John Desper

**Affiliations:** a Department of Chemistry, Kansas State University, Manhattan, KS 66506, USA; b Department of Chemistry and Biochemistry, Oberlin College, Oberlin, OH 44074, USA

**Keywords:** crystal engineering, hydrogen bonds, halogen bonds, synthons, electrostatic potentials

## Abstract

As halogen bonds gain prevalence in supramolecular synthesis and materials chemistry, it has become necessary to examine more closely how such interactions compete with or complement hydrogen bonds whenever both are present within the same system. As hydrogen and halogen bonds have several fundamental features in common, it is often difficult to predict which will be the primary interaction in a supramolecular system, especially as they have comparable strength and geometric requirements. To address this challenge, a series of molecules containing both hydrogen- and halogen-bond donors were co-crystallized with various monotopic, ditopic symmetric and ditopic asymmetric acceptor molecules. The outcome of each reaction was examined using IR spectroscopy and, whenever possible, single-crystal X-ray diffraction. 24 crystal structures were obtained and subsequently analyzed, and the synthon preferences of the competing hydrogen- and halogen-bond donors were rationalized against a background of calculated molecular electrostatic potential values. It has been shown that readily accessible electrostatic potentials can offer useful practical guidelines for predicting the most likely primary synthons in these co-crystals as long as the potential differences are weighted appropriately.

## Abstract 2

An extensive structural study on hydrogen and halogen bonding in co-crystals has helped clarify the competition and balance between them in a practical supramolecular synthesis.

## 1. Introduction   

Practical synthetic crystal engineering requires the ability to organize and connect molecular building blocks into desired solid-state motifs and architectures. Such endeavors rely on site-specific intermolecular interactions that facilitate the preparation of homomeric constructions as well as of heteromeric co-crystals *via* selective and hierarchical self-assembly. To develop robust, versatile supramolecular synthetic strategies, we need more information about the relative importance of two of the most useful non-covalent synthetic tools; hydrogen bonds (HBs) and halogen bonds (XBs).

The nature of the hydrogen bond, and its role in structural chemistry, has been extensively documented since the early twentieth century. Pauling devoted considerable attention to ‘hydrogen bonding’ in his seminal book from 1939 entitled *Nature of the Chemical Bond* (Pauling, 1960 ▸), and 20 years later, Piementel and McLellan summarized most of the available experimental data and relevant theoretical interpretations in ‘The Hydrogen Bond’ (Pimentel & McClellan, 1960 ▸). The abundance of papers on this topic has, almost inevitably, created occasional confusion regarding vocabulary as well as of the fundamentals of this interaction. It is interesting to note then that the most recent attempt by IUPAC (Arunan *et al.*, 2011 ▸) at unifying the language and terminology by which hydrogen bonding can be defined comes almost a century after Latimer and Rodebush proposed the concept of hydrogen bonding without actually using the term itself (Latimer & Rodebush, 1920 ▸). The basis of the IUPAC report is a broad analysis of the relevance and magnitude of the physical forces that drive hydrogen bonding and the dominant contribution in most hydrogen-bond interactions is the electrostatic component. However, the hydrogen bond is partially covalent in nature (McWeeny, 1979 ▸; Del Bene, 1970 ▸), and induction and dispersion, in addition to exchange correlation from short range repulsion, all have to be considered in order to fully appreciate the complexity of this chemical bond (Dykstra & Lisy, 2000 ▸; Umeyama & Morokuma, 1977 ▸). The IUPAC team also used crystallographic data in order to find unique bond lengths, angles and energies characteristic of hydrogen bonding. However, since it was deemed difficult to choose definitive hydrogen-bond distances (Raghavendra *et al.*, 2006 ▸; Klein, 2006 ▸) or energies (Pauling, 1960 ▸; Jeffrey & Saenger, 1991 ▸; Desiraju & Steiner, 1999 ▸), the linearity of a hydrogen bond was identified as the ‘discriminative attribute’ (Elghobashi & González, 2006 ▸). Spectroscopic data were also examined to find characteristic IR stretches and NMR shifts which would commonly accompany hydrogen bonds (*e.g.* frequent red-shift of *X*—H bands in the IR (Scheiner, 1997 ▸; Badger & Bauer, 1937 ▸) and a down-field shift in NMR (Hobza & Havlas, 2000 ▸)). However, alternative interpretations and views remain as to whether these spectroscopic methods produce consistent changes in response to the influence of hydrogen-bond interactions (Scheiner & Kar, 2002 ▸; Joseph & Jemmis, 2007 ▸). The efforts by the IUPAC task force clearly demonstrate that this topic is still hugely important and very complex.

Following closely behind the hydrogen bond, the halogen bond was highlighted as a viable non-covalent interaction some 60 years ago by Hassel (Hassel, 1970 ▸). It subsequently went through a rather quiet patch until Metrangolo and Resnati rejuvenated this field through a number of key articles (Metrangolo *et al.*, 2005 ▸). The halogen bond displays many fundamental similarities to the hydrogen bond, and it has been dissected and debated recently in ways that are very reminiscent of the way in which hydrogen bonding has been described. This attention to halogen bonding is fully justified given its importance in supramolecular synthesis, materials chemistry, biological systems and drug design (Bauzá *et al.*, 2011 ▸; Sarwar *et al.*, 2010 ▸). Halogen bonds are also ‘tunable’ through covalent modifications to the molecule on which the donor sites are found (Riley & Hobza, 2008 ▸, 2011 ▸). Electron-withdrawing groups facilitate the redistribution of electron density away from the tip of the halogen atom, thus making it more electropositive and a more effective halogen-bond donor. However, electrostatic forces are not solely responsible for defining the halogen bond as dispersion and induction also play a role (Jeziorski *et al.*, 1994 ▸), which means that the debate about the nature and strengths of different halogen-bond interactions is remarkably similar to that which has accompanied the hydrogen bond (Řezáč *et al.*, 2012 ▸; Riley & Hobza, 2013 ▸).

The question is, where does all this information leave the practitioner of synthetic crystal engineering? Hydrogen bonds and halogen bonds are complicated and subtle, directional yet reversible, chemical bonds, so how do we develop strategies that fully utilize the synthetic possibilities that these interactions offer, without having to resort to a serendipitous supramolecular combinatorial approach? One way of getting some answers may be through systematic structural studies where relatively simple custom-designed probe molecules, equipped with potentially competing hydrogen- and halogen-bond donor sites are introduced to a series of molecules decorated with different acceptor sites. By examining the structural outcome of a sufficient number of experiments, it may be possible to identify some of the finer details in the structural landscape that surrounds competing (or complementary) hydrogen and halogen bonds.

Studies that clearly address the balance between HBs and XBs are still quite unusual, but Desiraju and co-workers examined supramolecular synthons created through aniline–phenol interactions which included an analysis of the role played by secondary halogen bonds and π–π interactions (Mukherjee & Desiraju, 2014 ▸). Bruce and co-workers examined the outcome of reactions between 4-halo-tetrafluorophenols, which can act as both XB and HB donors, and a series of amines, and found that in each of the 11 structures that were reported (eight iodo- and three bromo-based donors) the outcome was a salt which was dominated by charge-assisted N—H^+^⋯O^−^ (phenolate) hydrogen bonds (Takemura, McAllister, Hart *et al.*, 2014 ▸). The loss of the —OH moiety as a hydrogen-bond donor (due to deprotonation) made it difficult to draw any conclusions about the possible competition between XB and HB donor sites. In another study with 4-iodotetrafluorobenzoic acid, 4-iodotetrafluorophenol and 4-bromotetrafluorophenol, Bruce and co-workers used dithiane as an acceptor molecule (Takemura, McAllister, Karadakov *et al.*, 2014 ▸) and found that careful co-former selection can lead to halogen-bond preference over hydrogen bonding consistent with an iodine basicity scale (Laurence *et al.*, 2011 ▸), but the study only had access to four crystal structures of neutral co-crystals. Finally, Aakeröy and co-workers showed that in molecules containing both pyridine and amino-pyrimidine sites, hydrogen bonds are responsible for the assembly of the primary structural motif while halogen bonds play supporting roles (Aakeröy *et al.*, 2009 ▸), and they also demonstrated that both hydrogen and halogen bonds can be used as simultaneous without structural interference if the main molecular recognition events are based upon a careful combination of geometric and electrostatic complementarity (Aakeröy *et al.*, 2011 ▸).

**Figure 1:**
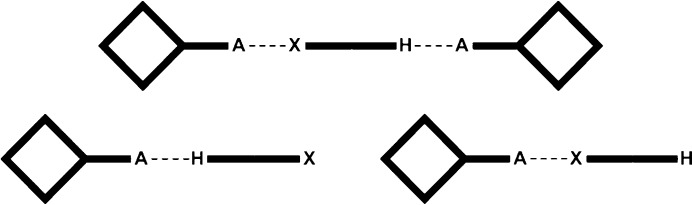
The three postulated outcomes of co-crystallizations with a monotopic acceptor (*X* = halogen-bond donor; H = hydrogen-bond donor, *A* = halogen-/hydrogen-bond acceptor).

**Figure 2:**
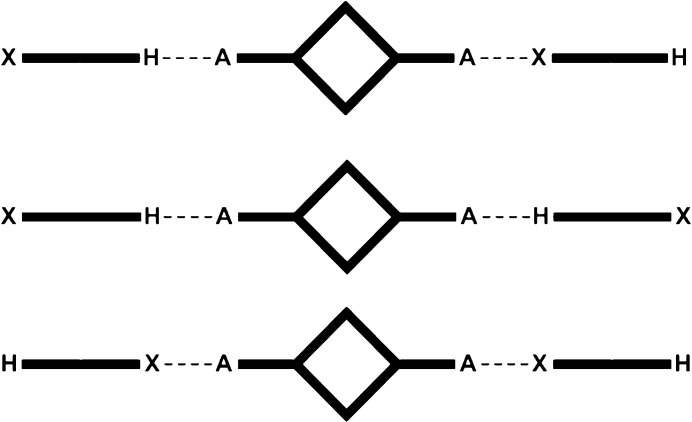
The three possible outcomes of co-crystallizations with a ditopic symmetric acceptor.

**Figure 3:**
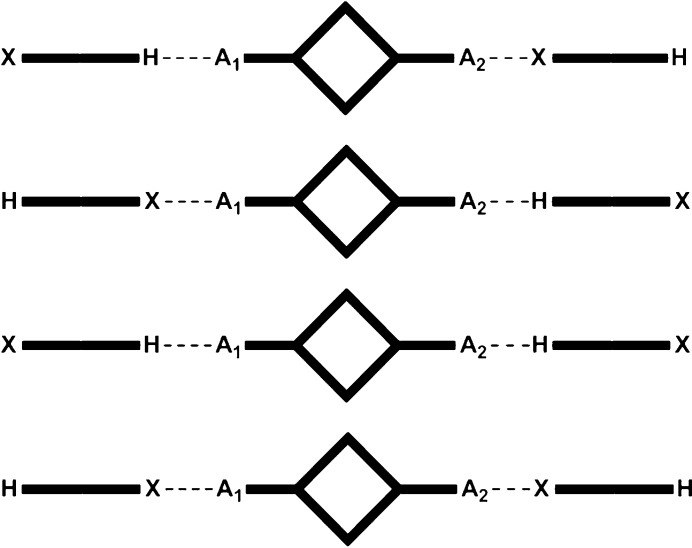
The four possible outcomes of co-crystallizations with a ditopic asymmetric acceptor (*A*_1_ = best acceptor; *A*_2_ = second best acceptor).

The goal of our study is primarily to utilize crystallographic data on co-crystals of a wide range of ditopic molecules, each carrying a hydrogen-bond donor and a halogen-bond donor, in order to determine which is the more effective supramolecular synthetic vector. Second, we want to explore a simplified electrostatic view of hydrogen/halogen-bond interactions as a versatile and *practical* method for *a priori* identifying the most likely or dominant synthon in a competitive molecular recognition event (the protocol and work plan are outlined in Figs. 1–3 ▸ ▸ ▸).

The first part of the study examines combinations of ditopic donors and monotopic acceptors with postulated outcomes presented in Fig. 1 ▸.

Second, ditopic symmetric acceptors were included in order to determine if the two donors were comparable in strength; this could be inferred if the HB donor formed an interaction with one acceptor site and the XB donor engaged with the other acceptor site, Fig. 2 ▸.

Finally, ditopic asymmetric acceptors were introduced, Fig. 3 ▸, to the HB/XB donors in order to probe how XB/HB donors would compete for acceptors sites offering electrostatic potential surfaces of different magnitudes (Etter, 1990 ▸).

**Figure 4:**
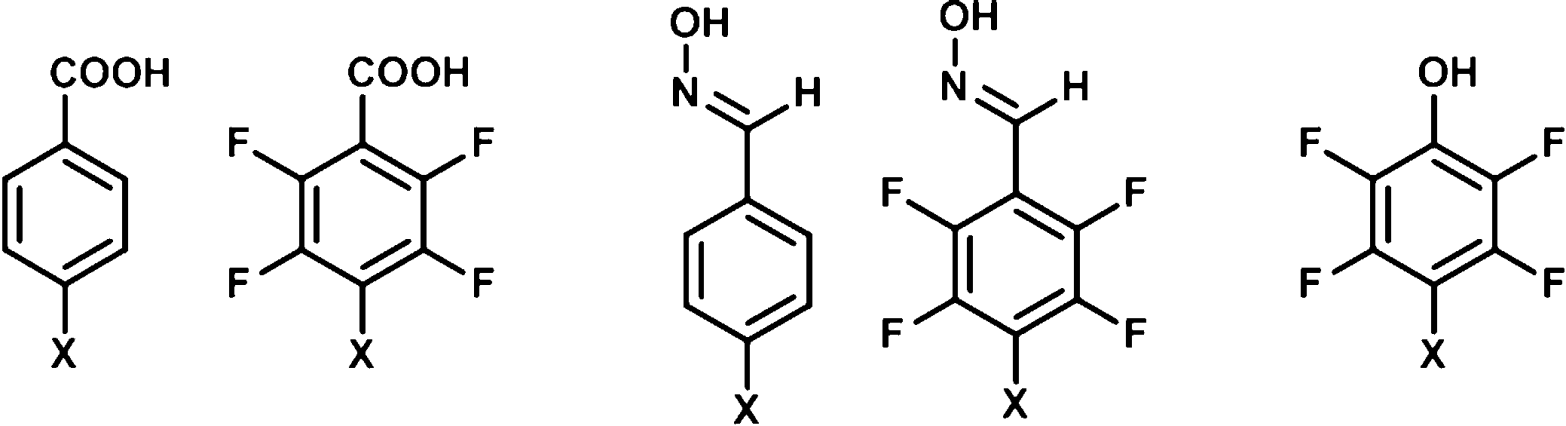
The hydrogen-/halogen-bond donors used in this study. *X* = I, Br.

In order to eliminate potentially misleading data resulting from possible solubility differences between hydrogen-bond donors and halogen-bond donors, the two donor sites were attached to the same molecular backbone, Fig. 4 ▸.

For the carboxylic acid and oxime donors, both the fluorinated and non-fluorinated versions of the iodo and bromo derivatives were used. However, the non-fluorinated phenolic ligands were not considered due to very low electrostatic potential values on the halogen-bond donors, indicating that they would not be competitive.

The results of this study may help us answer several key questions: *which is more effective, the hydrogen-bond donor or the halogen-bond donor?* Additionally, *when in direct competition with one another for acceptor molecules, what is the most likely outcome?* Even though numerous physical forces are needed to give a full account of either interaction, *is it possible to use readily accessible electrostatic potential surfaces as a way of ranking competing donors as well as predicting the most likely synthons?* The overall outcome of this study may help to formulate versatile and useful synthetic crystal engineering strategies that facilitate the directed assembly of specific solid-state motifs through predictable synthons.

## 2. Experimental   

### 2.1. Synthesis of ligands   

Unless otherwise noted, the donor and acceptor ligands, in addition to the solvents, used throughout these experiments were obtained commercially and without further purification. Melting points were taken using a Gallenkamp melting point apparatus (see Table 1 ▸).

2,3,5,6-Tetrafluoro-4-iodobenzoic acid (**IF_4_-COOH**) and 4-bromo-2,3,5,6-tetrafluorobenzoic acid (**BrF_4_-COOH**) were synthesized according to previously reported methods in the literature (Aakeröy *et al.*, 2011 ▸), whereas 4-iodobenzoic acid (**I-COOH**) and 4-bromobenzoic acid (**Br-COOH**) were purchased. (*E*)-2,3,5,6-Tetrafluoro-4-iodobenzaldehyde oxime (**IF­_4_-OX**), (*E*)-4-bromo-2,3,5,6-tetrafluorobenzaldehyde oxime (**BrF­_4_-OX**), (*E*)-4-iodobenzaldehyde oxime (**I-OX**) and (*E*)-4-bromobenzaldehyde oxime (**Br-OX**) were synthesized using a mechanochemical route (Aakeröy, Sinha *et al.*, 2012 ▸). 2,3,5,6-Tetrafluoro-4-iodophenol (**IF_4_-OH**) was synthesized by treating the corresponding pentafluoro­iodo­benzene with *tert*-butyl alcohol under reflux (Wen *et al.*, 1994 ▸) and 4-bromo-2,3,5,6-tetrafluorophenol (**BrF­_4_-OH**) was obtained commercially.

4-(Pyridine-4-yl)pyridine-1-oxide, pyrazine-1-oxide and 2,3,5,6-tetramethylpyrazine-1-oxide were synthesized according to literature methods (Aakeröy *et al.*, 2014*a* ▸). 5,6-Dimethyl-1-(pyridin-3-ylmethyl)-1*H*-benzo[*D*]imidazole, 5,6-dimethyl-1-(pyridin-4-ylmethyl)-1*H*-benzo[*D*]imidazole (Aakeröy, Desper & Smith, 2007 ▸) and 1-(pyridin-4-ylmethyl)-1*H*-benzo[*D*]imidazole (Aakeröy, Epa, Forbes, Schultheiss & Desper, 2013 ▸) were synthesized according to published procedures (see Table 2 ▸).

### 2.2. Electrostatic potential calculations   

Calculations of molecular electrostatic surface potentials were carried out using DFT with the B3LYP level of theory and a 6-31++G** basis set in vacuum. All calculations were carried out using *Spartan*’08 software. All molecules were geometry optimized with the maxima and minima in the electrostatic potential surface (0.002 e a.u.^−1^ isosurface) determined using a positive point charge in the vacuum as a probe. The numbers indicate the interaction energy (kJ mol^−1^) between the positive point probe and the surface of the molecule at that particular point. These numbers could be correlated to the electrostatic charges on the atoms with the negative number corresponding to negative charge and positive number corresponding to positive charge. The program automatically identifies the maximum/minimum points on the surface.

### 2.3. IR analysis   

**Figure 5:**
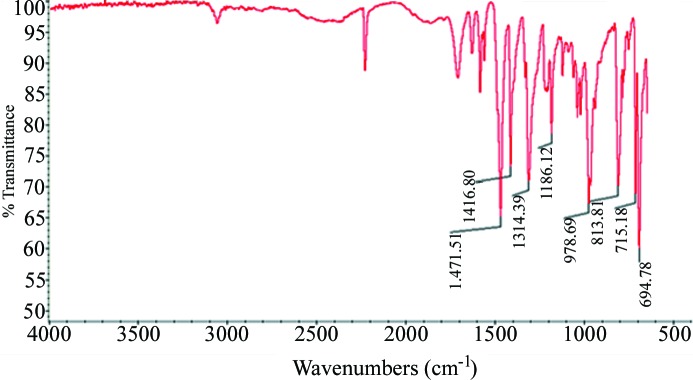
IR spectrum of **BrF_4_-COOH – 1**, showing O—H⋯N features at 1900 and 2450 cm^−1^.

The outcome of each attempted co-crystallization was analyzed using IR spectroscopy (Nicolet 380 FT-IR). Vibrational spectroscopy provides information about whether the two reactants have formed a heteromeric solid based on characteristic shifts or new key bands. For example, O—H⋯N(heterocycle) hydrogen bonds tend to produce two broad bands around 1900 and 2500 cm^−1^, Fig. 5 ▸.

### 2.4. Synthesis of co-crystals   

**Figure 6:**
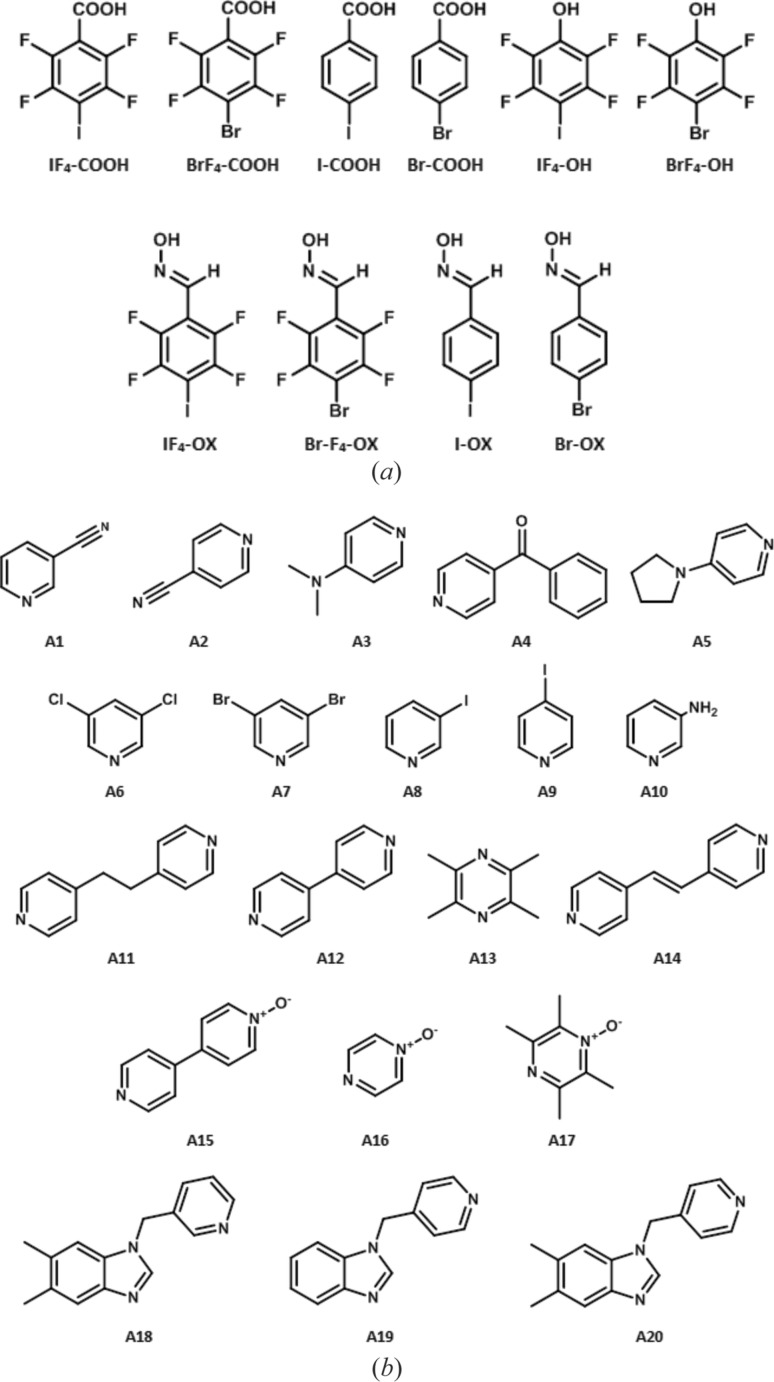
(*a*) Halogen-/hydrogen-bond donors, and (*b*) HB/XB acceptors.

Ten HB/XB ditopic donor molecules were combined with 20 different acceptors in a series of co-crystallization experiments, Fig. 6 ▸.

Stoichiometric amounts of the two reactants were mixed with a few drops of solvent and put through a solvent-assisted grinding protocol (James *et al.*, 2012 ▸; Aakeröy, Sinha *et al.*, 2012 ▸; Aakeröy, Chopade *et al.*, 2012 ▸). The details for the preparation of compounds that yielded crystals suitable for single-crystal X-ray diffraction are shown in Table 3 ▸.

### 2.5. Crystal structure analysis   

Crystallographic data can be found in the supporting information for all 24 structure determinations.

## 3. Results   

### 3.1. Electrostatic potential calculations   

The results for the ten HB/XB donors are displayed in Table 4 ▸, and the corresponding results for the 20 acceptors are included in the supporting information.

### 3.2. IR analysis   

Table 5 ▸ describes the outcomes of all 200 (10 × 20) attempted co-crystallizations as established by IR spectroscopy. The relative success rate for each donor, as well as for each acceptor, is also given.

Table 5 ▸ has been split into columns in order to emphasize the relationship between the different halogen-bond donors. For example, the first two columns show the fluorinated iodo- and bromo-species of benzoic acid, whereas the two columns to their right show the non-fluorinated analogues. This arrangement highlights the percent success for each donor type. It can be seen that in every case, the iodo-donor has an equivalent or higher percentage success than its analogous bromo-donor. Furthermore, the fluorinated analogues are more successful at co-crystal formation than their non-fluorinated counterparts, which is in agreement with the electrostatic potential values on the HB and XB donors, as shown at the top of the table.

### 3.3. Co-crystal results   

Over half of the 200 different donor:acceptor reactions carried out produced co-crystals and 24 of them yielded crystals suitable for single-crystal X-ray diffraction. Each acceptor can be placed in one of three categories: monotopic, ditopic symmetric and ditopic asymmetric. The possible connectivities and stoichiometries of the resulting supramolecular assemblies were described in Figs. 1–3 ▸ ▸ ▸. The results from the 24 new crystal structures are summarized in Tables 6–8 ▸ ▸ ▸.

Detailed crystallographic data has been included in the supporting information and deposited with the CCDC (1059404–1059416, 1059418–1059428), but relevant information about the primary hydrogen and halogen bonds is shown in Table 9 ▸. During the course of this study we were also able to isolate the structures for **IF4-OH--5** (Takemura, McAllister, Hart *et al.*, 2014 ▸) and **IF4-OH--12** (Takemura, McAllister, Karadakov *et al.*, 2014 ▸), but since they were recently reported by Bruce and co-workers, we have not included them in our results and will instead examine them as part of the discussion.

## 4. Discussion   

The 24 crystal structures were analyzed and classified according to acceptor type in order to elucidate any patterns of behavior regarding the competition between hydrogen and halogen bonds.

### 4.1. Monotopic acceptors   

**Figure 7:**
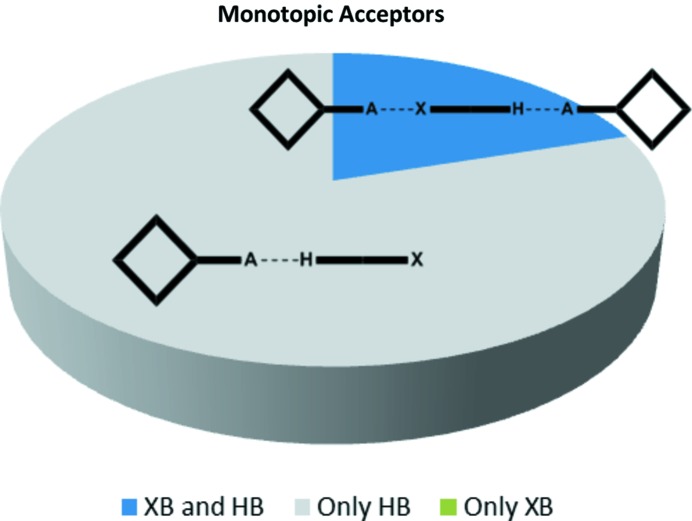
Distribution of motifs with monotopic acceptors.

**Figure 8:**
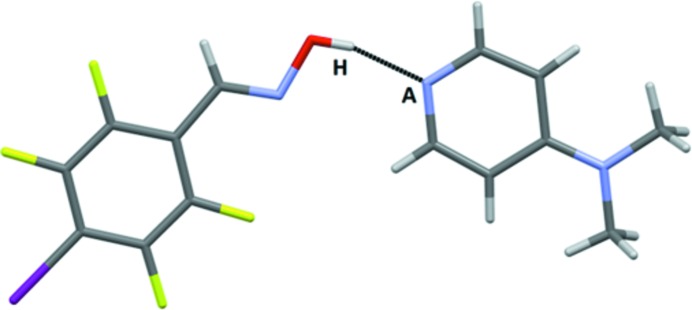
The main interaction in the crystal structure of **IF_4_-OX – 3** (*A* = acceptor, H = hydrogen-bond donor).

Five crystal structures were obtained with monotopic acceptors and the predominant outcome (4/5) was a co-crystal in a 1:1 stoichiometry assembled from hydrogen bonds with no discernable contributions from halogen bonds (Fig. 7 ▸). Three of the four representatives in this group (**IF_4_-OX – 3**, **Br-OX – 5** and **Br-COOH – 3**) displayed near-identical behavior (as postulated in Fig. 1 ▸, bottom left) with the two reactants held together by near-linear hydrogen bonds resulting in 1:1 dimeric species with no evidence of proton transfer, Fig. 8 ▸.

**Figure 9:**
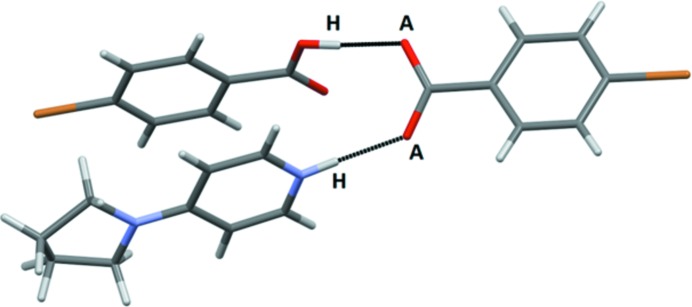
The salient intermolecular features in the crystal structure of 4-pyrrolidinopyridinium 4-bromobenzoate 4-bromobenzoic acid (1:1:1) (*A* = acceptor, H = hydrogen-bond donor).

However, in the fourth representative of this group, **Br-COOH – 5**, the outcome was somewhat different, even though only hydrogen bonding was noted as the structure-directing interactions. As a result of proton transfer from 4-bromobenzoic acid to 4-pyrrolidinopyridine (Fig. 9 ▸), an organic salt was created containing a carboxylate moiety as the key acceptor site. In addition to the benzoate:pyridinium ions, the lattice also included one equivalent of 4-bromobenzoic acid. The pyrrolidinium ring is disordered, and the carboxylate site forms two hydrogen bonds, O—H⋯C—O and N—H⋯C—O.

The presence of an ‘extra’ neutral molecule in pyridinum carboxylates is not unexpected as it has been demonstrated (Aakeröy, Fasulo & Desper, 2007 ▸) that close to 40% of organic carboxylate salts appear either as solvates/hydrates or with an additional neutral acid molecule in the crystal lattice. The likely explanation for this behavior is that a carboxylate moiety represents a powerful charge-assisted two-atom hydrogen-bond acceptor site which is not readily satisfied by a single hydrogen-bond donor, thus making it necessary to bring in a ‘free’ carboxylic acid or a suitable solvent molecule. In contrast, the charge distribution around a neutral carboxylic acid makes it a less powerful or demanding hydrogen-bond acceptor site. Strictly speaking, **Br-COOH – 5** may not fit exactly with any of the postulated outcomes in Fig. 1 ▸, but since no halogen bonding was observed, it belongs in the category of structures of monotopic acceptors where hydrogen-bonding dominates over halogen bonding.

**Figure 10:**
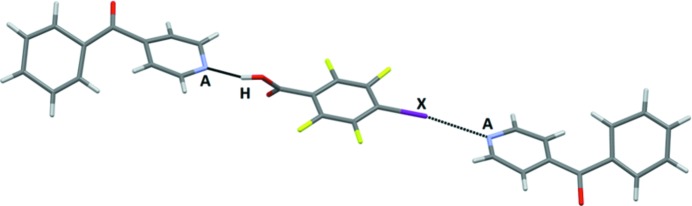
The trimeric supermolecule in the crystal structure of **IF_4_-COOH – 4** (*A* = acceptor, H = hydrogen-bond donor, *X* = halogen-bond donor).

In the remaining crystal structure with a monotopic acceptor, both halogen bonds and hydrogen bonds participate in the structure-directing process. The crystal structure of tetrafluoro-4-iodobenzoic acid 4-benzoylpyridine (**IF_4_-COOH – 4**) displays interactions involving both the carboxylic acid and the activated iodine atom, and the outcome is a trimeric supermolecule in a 1:2 ratio, Fig. 10 ▸ (as postulated in Fig. 1 ▸). Note that we are considering 4-benzoylpyridine as a monotopic species since ketones are generally regarded as very poor acceptor sites and compared to the capability of a pyridyl moiety, it is reasonable to classify benzoylpyridine as a monotopic acceptor.

Based on the five structures with monotopic acceptors, it seems that hydrogen bonding is marginally favored (we found no system when halogen bonds were present and hydrogen bonds were absent). However, it should be noted that in three of the four structures where hydrogen bonding was dominant, the potential halogen-bond donors were not activated through the presence of electron-withdrawing groups or an adjacent *sp*-hybridized C atom. On the other hand, in the case where hydrogen bonds and halogen bonds were present simultaneously, the latter were represented by strongly activated iodine atoms; **IF_4_-COOH**. These observations are discussed in detail later in the context of calculated molecular electrostatic potential values. It should be noted that the crystal structure of **IF4-OH – 5** has been previously reported by Bruce and co-workers (Takemura, McAllister, Hart *et al.*, 2014 ▸) (CCDC code: BIYFOG). The primary motifs in that structure are not the same as was found in **IF_4_-COOH-4**, due to the deprotonation of the hydroxyl group. The phenolate site has become the sole acceptor site and acts as a bifurcated acceptor to a charge-assisted N—H^+^ hydrogen bond and a C—I halogen bond. The bifurcated XB/HB interaction is almost symmetric with both C—O⋯H—N bond C—O⋯I bond angles close to 131°. The similarity in bond angles may indicate that the two interactions are very comparable in importance and that the two donors are equally competitive for the most prominent charge-rich regions around the phenolate oxygen atom.

### 4.2. Ditopic symmetric acceptors   

**Figure 11:**
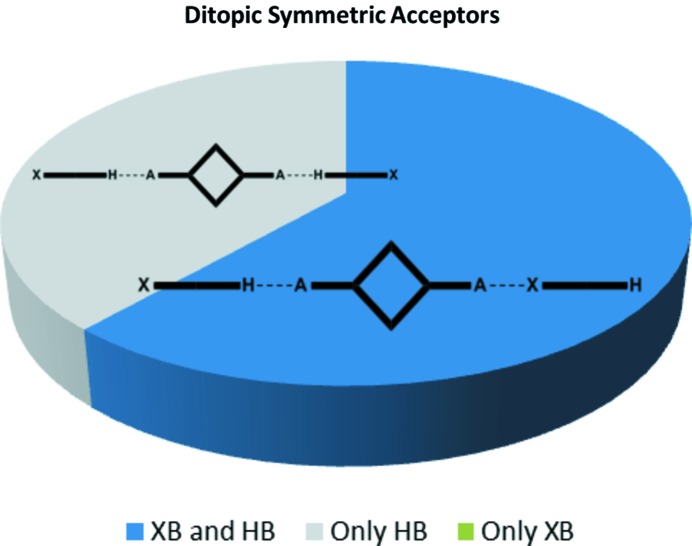
Distribution of motifs with ditopic symmetric acceptors.

Co-crystallizations involving ditopic molecules with two equivalent acceptor sites produced 13 crystal structures. We anticipated three different modes of assembly as shown in Fig. 2 ▸: hydrogen bonds at both sites, halogen bonds at both sites or a halogen bond at one end and a hydrogen bond at the other end of the acceptor, producing an infinite one-dimensional chain. In eight co-crystals both donor types were involved in the assembly of supramolecular infinite chains, and in the remaining five structures both sides of the acceptor form a hydrogen bond, Fig. 11 ▸. There was no instance where a halogen bond was solely responsible for the co-crystal assembly.

**Figure 12:**
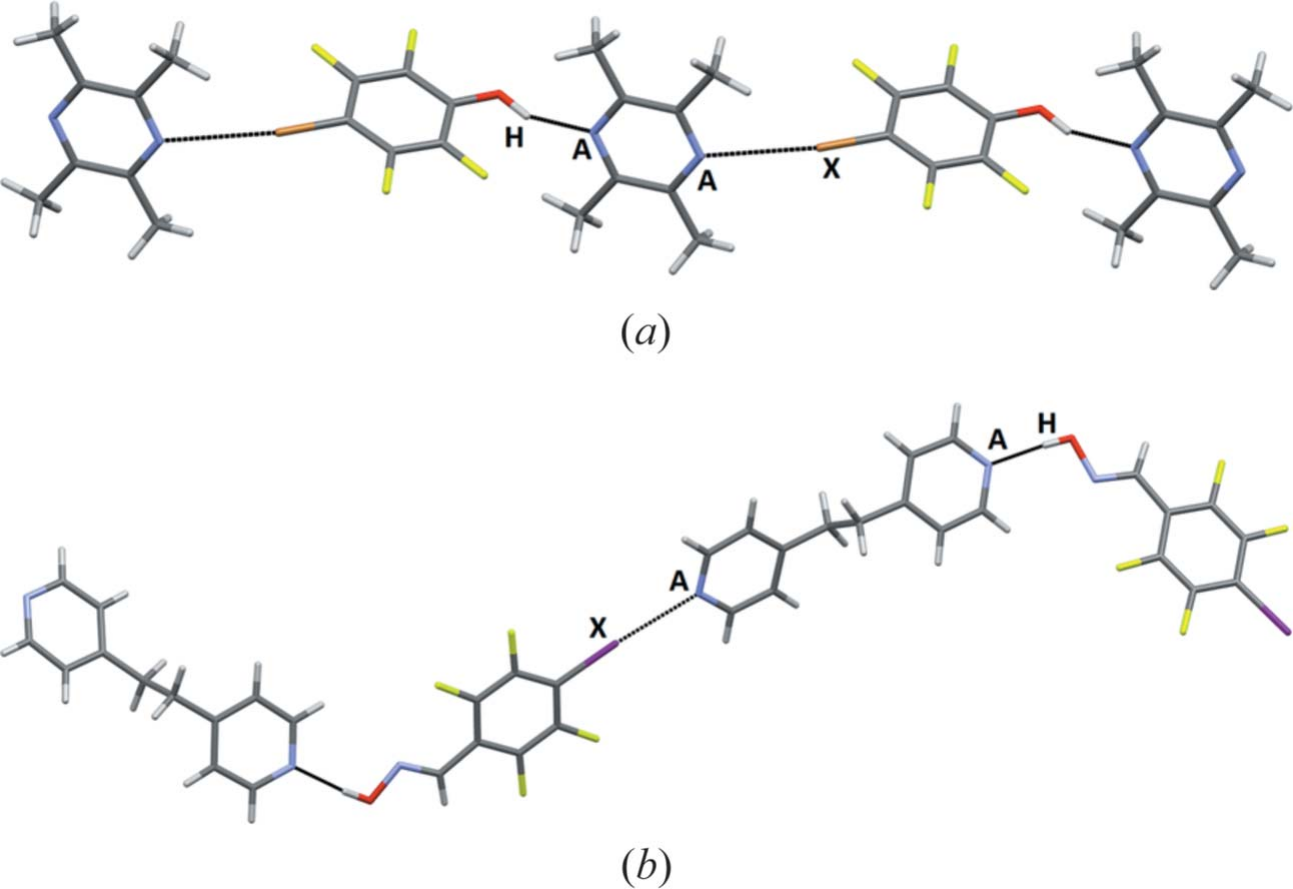
Primary interactions in the crystal structures of (*a*) **BrF_4_-OH – 13** and (*b*) **IF_4_-OX – 11** (bottom) (*A* = acceptor, H = hydrogen-bond donor, *X* = halogen-bond donor).

The infinite chains resulting from alternating donor and acceptor molecules are all very similar in terms of connectivity and geometry, Fig. 12 ▸ (in some cases the HB/XB donor molecule was disordered over two positions).

**Figure 13:**
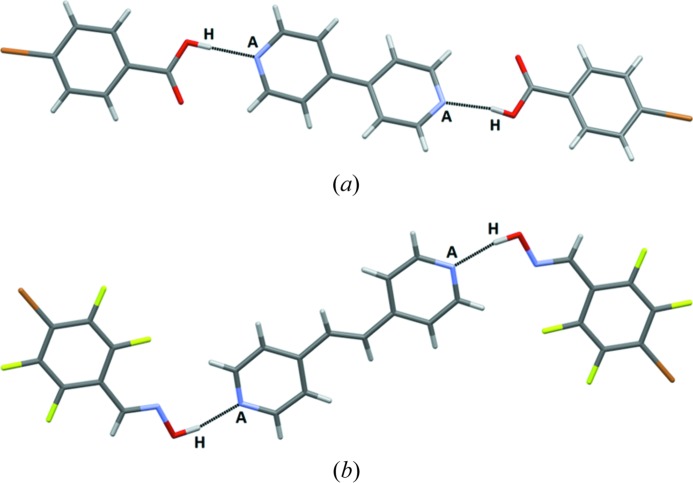
Supramolecular trimers in the structures of (*a*) **Br-COOH – 12** and (*b*) **BrF_4_-OX – 14** (*A* = acceptor, H = hydrogen-bond donor).

The second assembly type, found in five of the 13 structures with ditopic acceptors, in which only hydrogen bonding is observed, effectively leads to discrete supramolecular trimers, Fig. 13 ▸, with none of the main acceptor moieties engaged in a halogen bond.

The five structures where only hydrogen bonds appeared all involved bromo-substituted donors (**Br-COOH**, **BrF_4_-OX** and **BrF_4_-OH**). The lower polarizability of bromine compared with that of iodine clearly puts the XB donor at a significant disadvantage. Most of the eight chain-like motifs utilized an iodine-based HB donor (**I-COOH**, **IF_4_-OX**, **IF_4_-COOH**) even though some bromo-substituted donor molecules did produce a C—Br⋯*A* halogen bond alongside the HB donor, as long as the aromatic backbone was decorated with F atoms to activate the XB donor (as in **BrF_4_-OH** and **BrF_4_-COOH)**.

### 4.3. Ditopic asymmetric acceptors   

**Figure 14:**
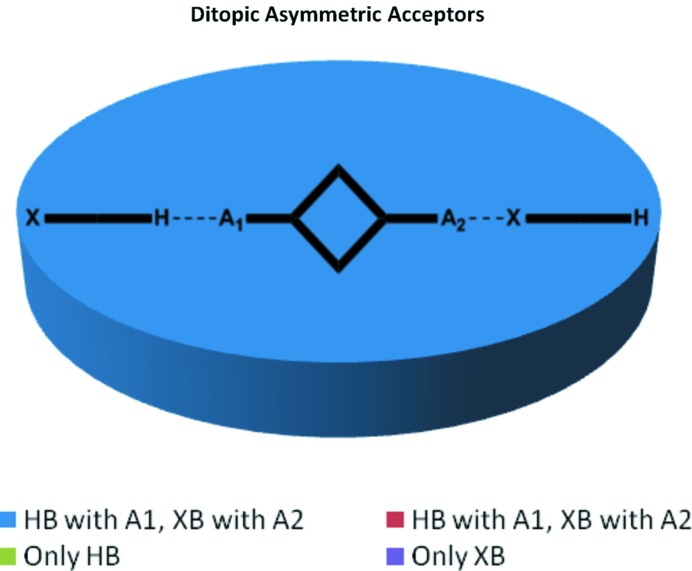
Distribution of motifs with ditopic asymmetric acceptor ligands.

**Figure 15:**
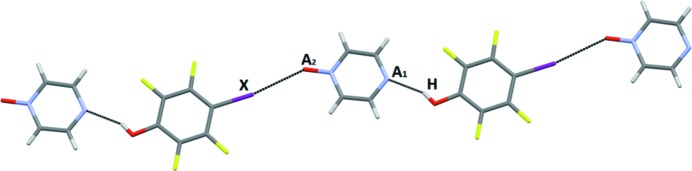
One-dimensional chains in the crystal structures of tetrafluoro-4-iodophenol pyrazine-1-oxide (*A*_1_ = best acceptor, *A*_2_ = second-best acceptor, H = hydrogen-bond donor, *X* = halogen-bond donor).

The final selection of co-crystals contained a ditopic molecule with two acceptor sites with different calculated electrostatic potentials (Fig. 14 ▸). The combination of these acceptors with the HB/XB donors could give rise to four possible scenarios, Fig. 3 ▸. Either the HB donor interacts with the stronger acceptor, leaving the XB donor to interact with the weaker, or *vice versa*. Alternatively, only one of the two donor types engage with both acceptors. Six crystal structures were obtained in this group but two of them, (IF_4_-COOH—2 and IF_4_-COOH—16), displayed disorder such that any assignment of binding preference could not be made. The four remaining structures were obtained with two different acceptor molecules, pyrazine-mono-*N*-oxide (**16**) and 4-CN-py (**2**). In the crystal structure of the co-crystal of the former, the HB donor interacts with the better acceptor and the XB donor interacts with the second best acceptor (ranking based upon electrostatic potentials (Aakeröy *et al.*, 2014*b* ▸; Aakeröy, Baldrighi *et al.*, 2013 ▸; Aakeröy, Chopade & Desper, 2013 ▸; Aakeröy, Epa, Forbes & Desper, 2013 ▸; Aakeröy, Epa, Forbes, Schultheiss & Desper, 2013 ▸; Aakeröy, Wijethunga & Desper, 2015 ▸) and keeping in mind that the potential on the *N*-oxide has to be distributed among several lone-pairs), Fig. 15 ▸.

**Figure 16:**
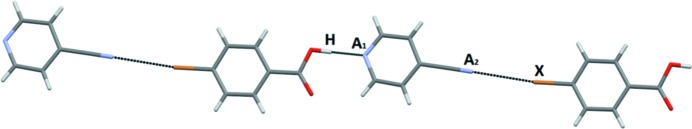
One-dimensional chains in the crystal structure of 4-bromobenzoic acid 4-cyanopyridine (*A*_1_ = best acceptor, *A*_2_ = second-best acceptor, H = hydrogen-bond donor, *X* = halogen-bond donor).

The three co-crystals with 4-CN-py displayed very consistent behavior; in each instance, the HB donor engaged with the py moiety, and the XB donor formed a halogen bond with the nitrile acceptor, Fig. 16 ▸.

**Figure 17:**
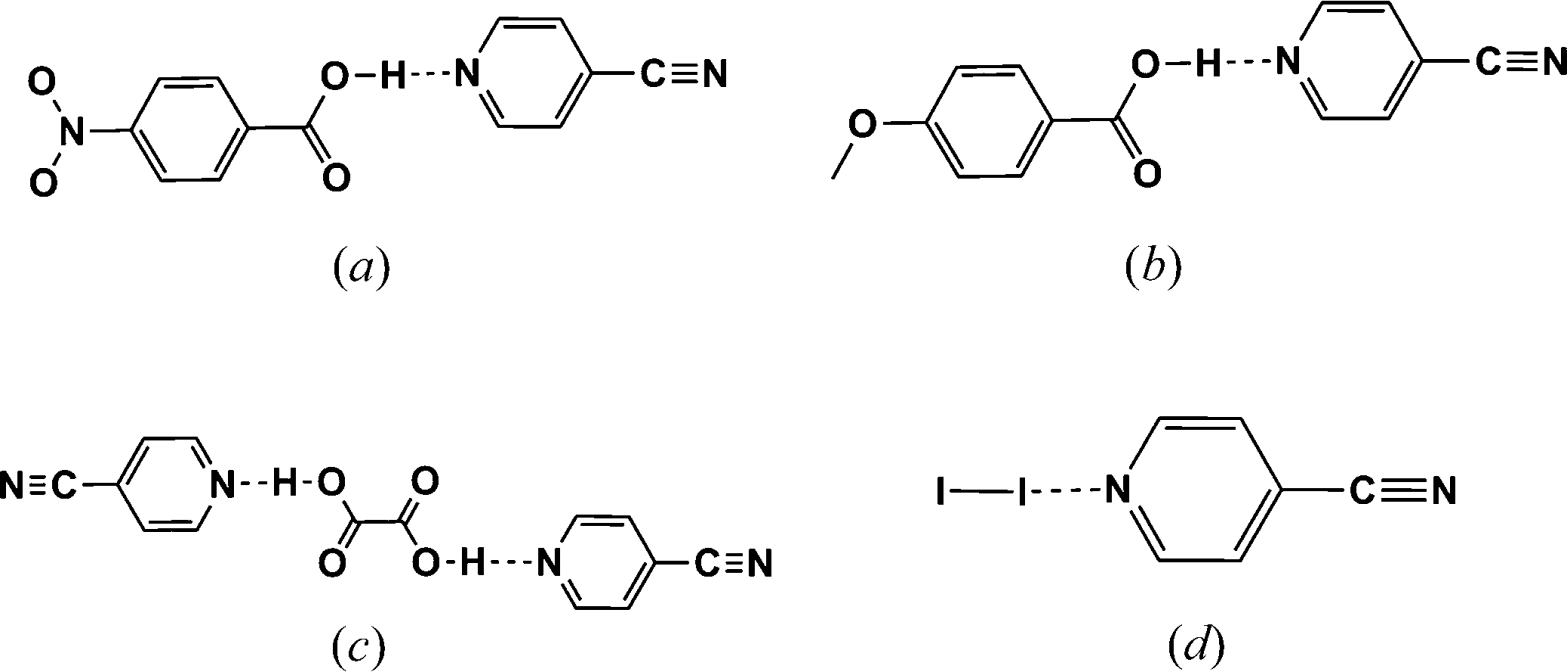
Common hydrogen-bond patterns (*a*)–(*b*) (Mukherjee & Desiraju, 2014 ▸) and (*c*) (Zheng, 2012 ▸) and halogen-bond pattern (*d*) (Bailey *et al.*, 1997 ▸) in co-crystals with 4-CN-pyridine.

We were surprised to note, however, that the DFT calculations indicated that the C=N group should be ranked as a better acceptor site than the py moiety as the calculated electrostatic potentials were −159 and −145 kJ mol^−1^, respectively. This ranking, (C=N) > (py), certainly seems counterintuitive, especially when considering extensive crystallographic data on reported co-crystals with 4-cyanopyridine; an analysis of existing relevant data clearly shows that the pyridine moiety is the preferred acceptor site. A few examples of motifs displayed by representative crystal structures are shown in Fig. 17 ▸.

Ultimately, this particular asymmetric acceptor must be examined in more detail with competing XB and HB donor moieties on the same molecule. However, based upon extensive crystallographic data, we will, for the purpose of this study, assign a ranking of (py) > (C=N) as indicated by the symbols *A*_1_ and *A*_2_, respectively in Fig. 16 ▸. The analysis presented in Fig. 14 ▸ is also based upon the same assignment.

Theoretical electrostatic potential calculations are known to offer valuable information about the relative strength of hydrogen bonds and halogen bonds (Murray & Politzer, 1991 ▸; Murray *et al.*, 1990 ▸), and our results also indicate that a relatively simple electrostatic description of such interactions provide a useful tool for predicting the most likely *practical* supramolecular outcome, even in relatively complex systems with multiple binding possibilities. An advantage of this simplistic approach for practical co-crystal synthesis is that the ranking of the different donors and acceptors can be achieved using readily available computational tools. It can be seen from Table 4 ▸ that the electrostatic potential value on the HB donor is significantly higher than on the halogen bond site, and this holds true for all ten ditopic donors. However, it is not possible to make a prediction of the outcome purely based upon electrostatic potentials when the system under consideration contains both XB and HB donors. Although the expected relative importance of hydrogen-bond donor and halogen-bond donors can be ranked within each group based on electrostatic potentials, it does not mean that we can use the potential values in a direct comparison between the two different types of donor moieties.

However, the systematic study presented herein does offer some insight into how the potential values of competing HB and XB donors can be utilized as a tool for predicting structural outcomes. First, every one of the 24 co-crystals presented here displays hydrogen bonding as one of the primary stabilizing interactions, but not every structure contains an obvious structure-directing halogen bond. The crystal structures of monotopic and ditopic symmetric acceptors fall into two groups; those with halogen bonding (9/18), and those without (9/18). Second, a closer analysis of the electrostatic potential values on each of the halogen bond donors in these systems showed that those structures with halogen bonding present had an average potential on the XB donor of 146 kJ mol^−1^, whereas those without halogen bonding had an average potential of the XB donor of 107 kJ mol^−1^. Clearly, unless the XB donor is sufficiently electrophilic, it will not match the structural impact of the competing HB donor.

**Figure 18:**
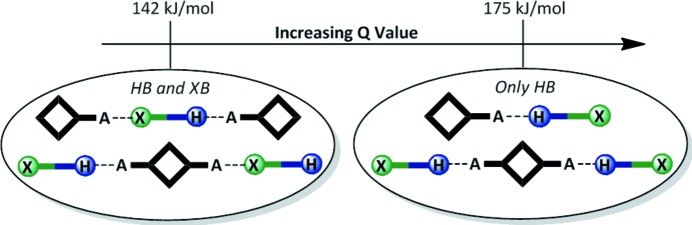
Correlation between difference in electrostatic potential (*Q* value) between HB and XB donor and structural outcome.

Another way of predicting the structural outcome in these systems involves using the relative differences in electrostatic potential of competing HB and XB donors. Therefore, *for the purpose of this study*, we define a single value, *Q*, as the difference in the electrostatic potential of the HB donor and the XB donor; *Q* = HB (potential) − XB (potential). The average *Q* value for the 11 structures that contained both hydrogen and halogen bonding (with monotopic or symmetric ditopic donors) was 142 kJ mol^−1^, whereas the average *Q* value for the nine structures that only displayed hydrogen bonding was 175 kJ mol^−1^. This underscores that the difference in electrostatic potential between competing sites can offer a good indication of what the outcome is likely to be in competitive supramolecular systems, Fig. 18 ▸.

If we were to rely on the average *Q* values as a way of estimating the outcome in the 20 structures with monotopic and symmetric ditopic acceptors, the correct primary structural features are predicting 89% of the time (in 16/18 structures). Only two outliers are observed, the first being the crystal structure of **IF_4_-OX – 3**, Fig. 8 ▸, where a hydrogen-bonded dimer was formed when we would have anticipated an HB/XB trimeric motif, with one donor molecule and two acceptors (the *Q* value in this case is 115 kJ mol^−1^). The second outlier among this group is the structure of **BrF_4_-OH – 13**, where the *Q* value for the donor is 189 kJ mol^−1^, and one would expect that HB would be formed exclusively resulting in a trimer. Instead, both the XB and the HB moieties act as donors and the result is an infinite chain, Fig. 12 ▸ (top).

**Figure 19:**
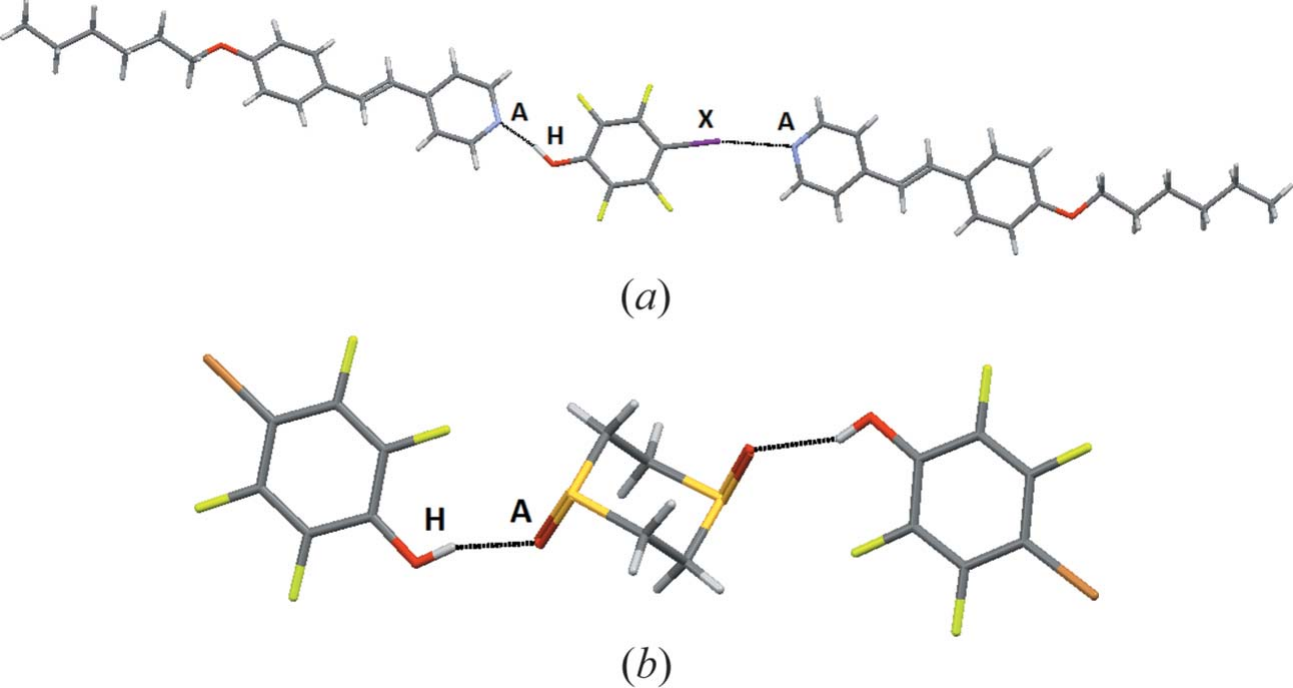
Supramolecular trimers observed in CSD structures with (*a*) **IF_4_-OH** (Präsang *et al.*, 2008 ▸) and (*b*) **BrF_4_-OH** (Takemura, McAllister, Karadakov *et al.*, 2014 ▸) (*A* = acceptor, H = hydrogen-bond donor, *X* = halogen-bond donor).

In the case of the interactions between a dual XB/HB donor molecule with either monotopic or symmetric ditopic acceptor molecules, we have been able to correlate the structural behavior with the relative difference in the electrostatic potential values of the two donor sites. In order to examine how well (or poorly) these *Q* values work for predicting the primary outcomes of co-crystallizations with XB/HB ditopic donor molecules and monotopic and ditopic acceptors, we found five structures in the CSD of direct relevance to this work. There are four neutral co-crystals with **IF_4_-OH** which has a *Q* value of 155 kJ mol^−1^ (TONMIT/TONMAL (Präsang *et al.*, 2008 ▸), HIZRIT/HIZROZ (Takemura, McAllister, Karadakov *et al.*, 2014 ▸)) and one co-crystal with **BrF_4_-OH**, which has a *Q* value of 190 kJ mol^−1^ (HIZREP (Takemura, McAllister, Karadakov *et al.*, 2014 ▸)), Fig. 19 ▸.

Based on the relative differences in electrostatic potentials for the two donors, one would expect the first group to contain both hydrogen and halogen bonds, since it is nearer to the average *Q* value of 142 kJ mol^−1^ exhibited in those cases. The latter structure would be expected to display only hydrogen bonds, since it exceeds the average *Q* value of 175 kJ mol^−1^ in which no XB exist. These are, in fact, the outcomes for each of the five crystal structures (Fig. 19 ▸). Even though there is still a relatively small amount of crystallographic data on co-crystals of molecules that contain one XB and one HB donor on the same molecular backbone, we have developed a simple electrostatic-based guideline for predicting the most likely *practical* outcome in systems with competing hydrogen bonds and halogen bonds. Once more relevant experimental data is added, the initial average *Q* values can be adjusted to better reflect the pattern preferences of a larger group of molecules. The work presented herein can offer a complement to studies that have examined connections and interrelationships between synthons, electron densities and structure or packing features in solids. For example, Hathwar and co-coworkers (Hathwar *et al.*, 2011 ▸) have proposed a *Supramolecular Synthon Based Fragments Approach* (SBFA) that relies on the robustness and modularity of the supramolecular synthons to provide transferability of charge-density-derived parameters for structural fragments, thereby providing a tool for accessing charge densities of unknown compounds. The SBFA approach has been validated against experimental charge density data in order to examine the reliability of this methodology (Dubey *et al.*, 2014 ▸).

The relationship between electron density and intermolecular bond energy has been examined for halogen bonds both theoretically (Amezaga *et al.*, 2010 ▸) and experimentally (Pavan *et al.*, 2013 ▸). Similarly, the nature and strength of hydrogen bonds have also been the subject of careful analyses using electron densities as a critical component (Jarzembska *et al.*, 2013 ▸) and such studies are not restricted to small molecules (Liebschner *et al.*, 2011 ▸). Furthermore, the balance between intermolecular interactions is obviously not always going to be dominated by hydrogen and halogen bonds and other forces, including dispersion, are always present to a greater or lesser extent (Maloney *et al.*, 2014 ▸).

In our study we have selected XB and HB donors–acceptors where steric hindrance is unlikely to play a role, but the importance of geometric factors for synthon reliability and crystal packing features has been highlighted through the use of long-range synthon Aufbau modules (LSAM) that carry the imprint of the synthons (Ganguly & Desiraju, 2010 ▸). Each LSAM can be characterized by specific geometries and relative orientations that may strongly influence the final assembly of the crystal lattice. This approach offers a complementary way of examining crystal assembly from individual molecules (or functional groups) to the final three-dimensional architecture and it may be particularly useful for constructing solids with specific unit-cell dimensions (Mukherjee *et al.*, 2014 ▸). The geometric disposition of chemical functionalities or binding sites can obviously influence the propensity for co-crystal formation and a multi-layered approach is especially necessary for rationalizing structures that defy expectations (Kaur *et al.*, 2015 ▸).

It is fair to say that sophisticated charge/electron-density studies remain non-routine and therefore a simplistic approach, based on extensive crystallographic information and readily accessible computational data as demonstrated in our work, can offer guidelines for how to predict key structural features in complex organic compounds with multiple co-existing synthons that may be of considerable practical value.

## 5. Conclusions   

This extensive structural study on the competition between hydrogen and halogen bonding in co-crystals has helped clarify the competition and balance between them in a practical supramolecular synthetic system. Building on a systematic co-crystal screen of 10 HB/XB donor molecules with 20 acceptors it has been shown that generally speaking hydrogen bonding is likely to be a more effective synthetic vector as a result of its presence in every one of the 24 structures obtained. However, halogen bonding is clearly also important for organizing molecules into well defined supramolecular motifs and extended architectures since such interactions appeared in 13 of the 24 structures. Whether a halogen bond appears alongside a hydrogen bond in any of the crystal structures herein or not is largely predicted upon the difference in electrostatic potential value between the HB donor and the XB donor (represented by the *Q* value). In structures of monotopic and symmetric ditopic acceptors where both XB and HB interactions were involved (9/18 occurrences) the average *Q* value was 142 kJ mol^−1^, whereas in the nine structures where only hydrogen bonding was present as a structure directing force, the average *Q* value was 175 kJ mol^−1^. We have deliberately avoided any discussions about how our results may or may not reflect the actual bond strengths of HB and XB interactions and instead simply focused on observed structural outcomes. The straightforward and readily applicable approach that comes out of this study for predicting the primary synthons is admittedly only based on electrostatics, but it nevertheless yields the correct synthons in 16 of the 18 structures. Obviously, further exceptions to our observations will arise, and it is clear that the structural landscape needs to be defined and examined with even greater resolution, but the information presented herein may offer a useful ‘rule-of-thumb’ for how the balance between potentially competing XBs and HBs will manifest itself in practical co-crystal synthesis.

## Supplementary Material

Crystal structure: contains datablock(s) IF4OX3, BrOX5, BrCO5, BrCO3, IF4CO4, ICO12, ICO11, BrF4OH13, BrF4CO11, IF4OX13, IF4OX11, IF4CO13, IF4CO12, BrCO11, BrCO12, BrF4OX14, BrF4OH12, BrF4OH11, IF4OH16, IF4OH2, IF4CO16, IF4CO2, BrCO2, BrF4OH2. DOI: 10.1107/S2052252515010854/lc5065sup1.cif

Experimental data: crystallography, synthesis. DOI: 10.1107/S2052252515010854/lc5065sup2.pdf

Structure factors: contains datablock(s) BrCO11. DOI: 10.1107/S2052252515010854/lc5065BrCO11sup3.hkl

Structure factors: contains datablock(s) BrCO12. DOI: 10.1107/S2052252515010854/lc5065BrCO12sup4.hkl

Structure factors: contains datablock(s) BrCO2. DOI: 10.1107/S2052252515010854/lc5065BrCO2sup5.hkl

Structure factors: contains datablock(s) BrCO3. DOI: 10.1107/S2052252515010854/lc5065BrCO3sup6.hkl

Structure factors: contains datablock(s) BrCO5. DOI: 10.1107/S2052252515010854/lc5065BrCO5sup7.hkl

Structure factors: contains datablock(s) BrF4CO11. DOI: 10.1107/S2052252515010854/lc5065BrF4CO11sup8.hkl

Structure factors: contains datablock(s) BrF4OH11. DOI: 10.1107/S2052252515010854/lc5065BrF4OH11sup9.hkl

Structure factors: contains datablock(s) BrF4OH12. DOI: 10.1107/S2052252515010854/lc5065BrF4OH12sup10.hkl

Structure factors: contains datablock(s) BrF4OH13. DOI: 10.1107/S2052252515010854/lc5065BrF4OH13sup11.hkl

Structure factors: contains datablock(s) BrF4OH2. DOI: 10.1107/S2052252515010854/lc5065BrF4OH2sup12.hkl

Structure factors: contains datablock(s) BrF4OX14. DOI: 10.1107/S2052252515010854/lc5065BrF4OX14sup13.hkl

Structure factors: contains datablock(s) BrOX5. DOI: 10.1107/S2052252515010854/lc5065BrOX5sup14.hkl

Structure factors: contains datablock(s) ICO11. DOI: 10.1107/S2052252515010854/lc5065ICO11sup15.hkl

Structure factors: contains datablock(s) ICO12. DOI: 10.1107/S2052252515010854/lc5065ICO12sup16.hkl

Structure factors: contains datablock(s) IF4CO12. DOI: 10.1107/S2052252515010854/lc5065IF4CO12sup17.hkl

Structure factors: contains datablock(s) IF4CO13. DOI: 10.1107/S2052252515010854/lc5065IF4CO13sup18.hkl

Structure factors: contains datablock(s) IF4CO16. DOI: 10.1107/S2052252515010854/lc5065IF4CO16sup19.hkl

Structure factors: contains datablock(s) IF4CO2. DOI: 10.1107/S2052252515010854/lc5065IF4CO2sup20.hkl

Structure factors: contains datablock(s) IF4CO4. DOI: 10.1107/S2052252515010854/lc5065IF4CO4sup21.hkl

Structure factors: contains datablock(s) IF4OH16. DOI: 10.1107/S2052252515010854/lc5065IF4OH16sup22.hkl

Structure factors: contains datablock(s) IF4OH2. DOI: 10.1107/S2052252515010854/lc5065IF4OH2sup23.hkl

Structure factors: contains datablock(s) IF4OX11. DOI: 10.1107/S2052252515010854/lc5065IF4OX11sup24.hkl

Structure factors: contains datablock(s) IF4OX13. DOI: 10.1107/S2052252515010854/lc5065IF4OX13sup25.hkl

Structure factors: contains datablock(s) IF4OX3. DOI: 10.1107/S2052252515010854/lc5065IF4OX3sup26.hkl

CCDC references: 1405186, 1405187, 1405188, 1405189, 1405190, 1405191, 1405192, 1405193, 1405194, 1405195, 1405196, 1059415, 1059416, 1059418, 1059419, 1059420, 1059421, 1059422, 1059423, 1059424, 1059425, 1059426, 1059427, 1059428

## Figures and Tables

**Table 1:** Melting points of synthesized ditopic donors

Donor	Observed melting point (C)	Literature data (C)
**IF_4_-COOH**	136139 dec.	140 dec. (Aakery *et al.*, 2011 ▸)
**BrF_4_-COOH**	130133	128130 (Aakery *et al.*, 2011 ▸)
**IF_4_-OX**	165167	165169 (Aakery, Sinha *et al.*, 2012 ▸)
**BrF_4_-OX**	138140	173175 (Aakery, Sinha *et al.*, 2012 ▸)
**I-OX**	101108	101103 (Aakery, Sinha *et al.*, 2012 ▸)
**Br-OX**	100105	110112 (Narsaiah Nagaiah, 2004 ▸)
**IF_4_-OH**	4750	4646.5 (Wen *et al.*, 1994 ▸)

**Table 2:** Melting points of synthesized acceptors

Acceptor	Observed melting point (C)	Literature data (C)
**A15**	170172	170171 (Aakery *et al.*, 2014*a* ▸)
**A16**	110113	113115 (Aakery *et al.*, 2014*a* ▸)
**A17**	98100	113115 (Aakery *et al.*, 2014*a* ▸)
**A18**	126131	150153 (Aakery, Desper Smith, 2007 ▸)
**A19**	98108	105110 (Aakery, Epa, Forbes, Schultheiss Desper, 2013 ▸)
**A20**	179184	182190 (Aakery, Desper Smith, 2007 ▸)

**Table 3:** Synthesis, melting points and crystal habit

*D* *A*	*D*:*A* ratio	Solvent†	Melting point (C)	Shape/color
**IF_4_-COOH2**	1:1	MeOH/CH_2_Cl_2_	125129	Colorless blocks
**IF_4_-COOH4**	1:4	EtOH/CH_2_Cl_2_	102104	Large colorless needles
**IF_4_-COOH12**	1:1	MeOH/trace CH_2_Cl_2_	165170 dec.	Colorless thin plates
**IF_4_-COOH13**	1:1	MeOH	138141 dec	Colorless rectangular prisms
**IF_4_-COOH16**	1:2	EtOAc/NitroMe	111115	Colorless blocks
**BrF_4_-COOH11**	1:1	Chloroform	137139	Off-white prisms
**I-COOH11**	1:1	MeOH/EtOH	190192	Opaque prisms
**I-COOH12**	1:2	MeOH/EtOH	179182	Colorless plates
**Br-COOH2**	1:4	MeOH	210221	Colorless, flat, large plates
**Br-COOH3**	1:4	EtOAc	151153	Colorless blocks
**Br-COOH5**	1:4	EtOAc	159162	Colorless, short, thin needles
**Br-COOH12**	1:2	MeOH	159164 dec.	Colorless blocks
**Br-COOH11**	1:2	MeOH/EtOH	140142	Colorless blocks
**IF_4_-OX3**	1:1	MeOH	9094 dec.	Yellow, wide needles
**IF_4_-OX11**	1:1	MeOH	135142	Opaque, rectangular prisms
**IF_4_-OX13**	1:1	EtOAc/NitroMe	133135	Colorless thin needles
**BrF_4_-OX14**	1:1	MeOH/EtOH	141145	Colorless long, thin needles
**Br-OX5**	1:1	MeOH	108112	Colorless, rectangular prisms
**IF_4_-OH2**	1:1	MeOH	9798 dec.	Light yellow needles
**IF_4_-OH16**	1:1	MeOH	102106 dec.	Large orange rectangular prism
**BrF_4_-OH2**	1:1	MeOH	100103 dec.	Light yellow needles
**BrF_4_-OH11**	1:1	MeOH	125130	Colorless, long needles
**BrF_4_-OH12**	1:1	MeOH	118119	Colorless, long, thin needles
**BrF_4_-OH13**	1:1	MeOH	119121 dec.	Flat colorless rectangular plate

† MeOH = methanol, EtOH = ethanol, CH_2_Cl_2_ = dichloromethane, EtOAc = ethyl acetate, NitroMe = nitromethane.

**Table 4:** Molecular electrostatic potential values for the HB/XB donors

Donor ligand	Hydrogen atom (kJmol^1^)	Halogen atom (kJmol^1^)
**IF_4_-COOH**	301.5	167.1
**BrF_4_-COOH**	288.3	139.1
**I-COOH**	266.9	112.8
**Br-COOH**	273.7	87.3
**IF_4_-OX**	273.8	158.9
**BrF_4_-OX**	279.0	127.8
**I-OX**	256.1	100.6
**Br-OX**	258.7	77.2
**IF_4_-OH**	304.8	149.6
**BrF_4_-OH**	315.3	125.8

**Table 5:** Outcome of co-crystal synthesis In this table (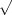) indicates a co-crystal and () indicates no reaction.

		Donors										
		**IF_4_-COOH**	**BrF_4_-COOH**	**I-COOH**	**Br-COOH**	**IF_4_-OX**	**BrF_4_-OX**	**I- OX**	**Br- OX**	**IF_4_-OH**	**BrF_4_-OH**	
XB potential (kJmol^1^)		167	139	113	87	159	128	101	77	150	126	
HB potential (kJmol^1^)		302	288	267	274	247	279	256	259	305	315	% Success
**Acceptors**	1	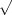	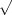		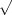	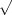	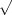			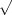	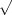	70
	2	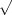	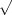	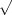	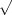	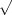	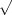	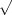		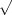	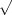	90
	3					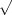	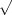	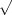	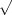		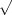	50
	4	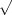								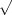	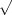	30
	5					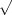	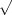	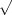	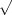		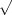	50
	6	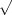	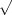									20
	7	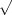	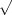							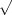		30
	8	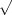		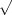		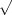				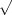	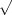	50
	9	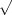	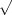	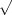	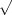	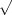	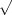	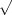		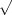	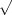	90
	10	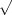				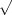		** 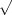 **				30
	11			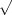	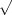	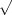	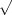	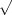			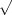	60
	12			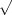	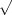	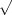	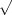			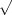	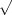	60
	13	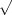	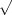			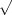				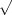	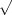	50
	14			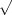	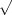	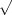	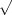		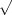	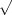	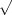	70
	15					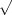	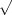	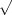	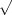	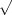	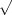	60
	16	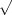	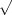	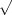	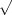	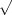		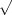				60
	17	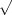	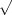				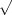		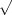	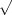		50
	18	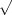		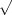	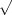				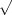	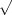		50
	19									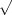	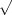	20
	20			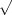		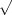	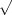			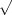	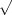	50
		12/20	8/20	9/20	8/20	14/20	11/20	8/20	6/20	14/20	14/20	
% Success		60	40	45	40	70	55	40	30	70	70	

**Table 6:** Monotopic acceptors (*X* = halogen-bond donor; H = hydrogen-bond donor; *A* = acceptor)

Crystal structures	Scheme	Codes
1/5	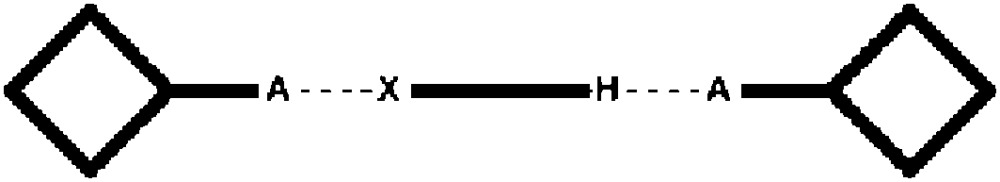	**IF_4_-COOH4**
4/5	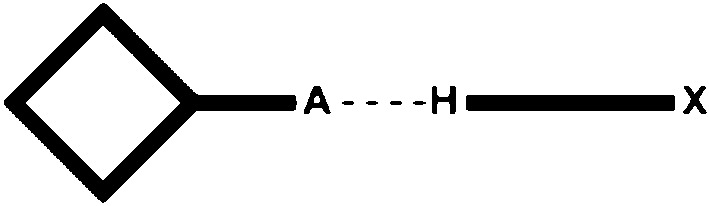	**IF_4_-OX3**
		**Br-OX5**
		**Br-COOH5**
		**Br-COOH3**
0	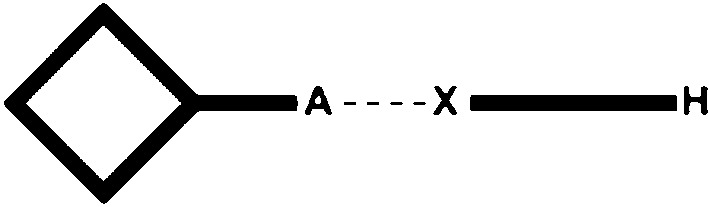	

**Table 7:** Ditopic symmetric acceptors (*X* = halogen-bond donor; H = hydrogen-bond donor; *A* = acceptor)

Crystal structures	Scheme	Codes
8/13	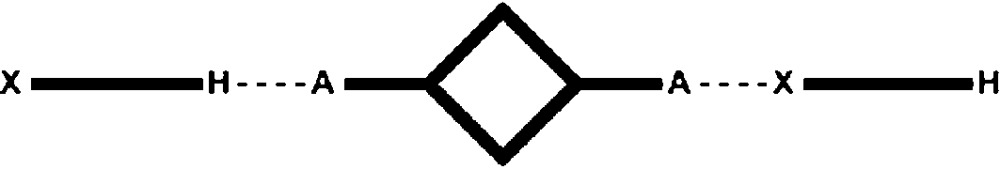	**I-COOH12**
		**I-COOH11**
		**BrF_4_-OH13**
		**BrF_4_-COOH11**
		**IF_4_-OX13**
		**IF_4_-OX11**
		**IF_4_-COOH13**
		**IF_4_-COOH12**
5/13	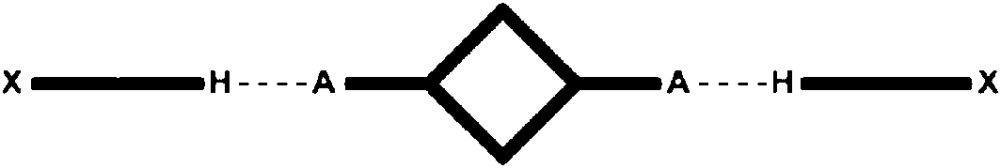	**Br-COOH11**
		**Br-COOH12**
		**BrF_4_-OX14**
		**BrF_4_-OH11**
		**BrF_4_-OH12**
0	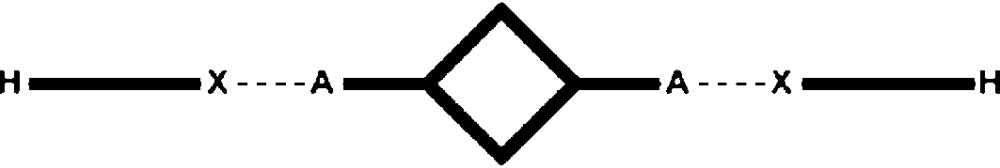	

**Table 8:** Ditopic asymmetric acceptors (*X* = halogen-bond donor; H = hydrogen-bond donor)

Crystal structures	Scheme	Codes
4/4	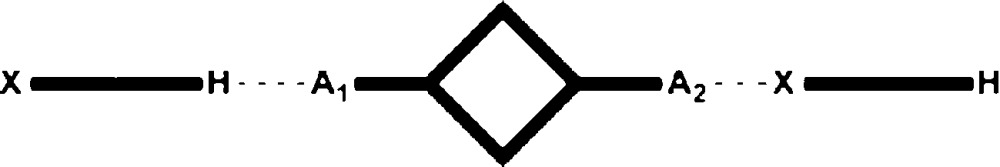	**IF_4_-OH16**
		**IF_4_-OH2**
		**Br-COOH2**
		**BrF_4_-OH2**
0	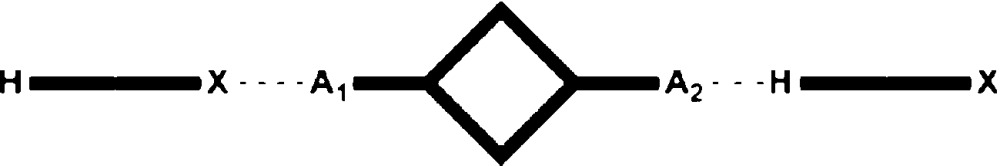	
0	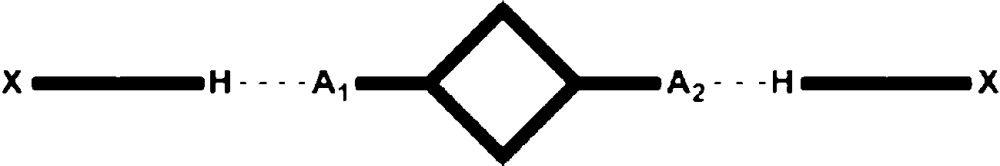	
0	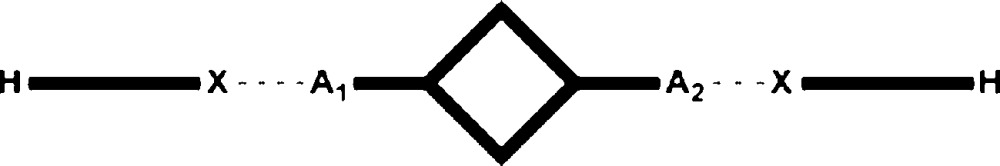	

The crystal structures of **IF_4_-COOH16** and **IF_4_-COOH2** were both disordered in such a way that no determination of binding preference of the HB and XB donor moieties could be made.

**Table 9:** Summary of hydrogen and halogen bond lengths () and angles ()

Code	HB distance () heavy atom*A*	HB angle ()	XB distance () *X* *A*	% van der Waals radii reduction	XB angle ()
**IF_4_-OX3**	2.649(3)	164.2			
**Br-OX5**	2.678(2)	171(3)			
**Br-COOH5**	2.509(3)	173(4)			
	2.690(3)	172(4)			
**Br-COOH3**	2.7077(16)	173.1(18)			
**IF_4_-COOH4**	2.531(16)	164	2.788(10)	21	173.7(5)
**I-COOH12**	2.666(9)	173.9	2.941(8)	17	177.8(3)
**I-COOH11**	2.681(12)	173.1	2.950(8)	16	176.2(7)
**BrF_4_-OH13**	2.659(3)	152(4)	3.017(2)	11	168.89(11)
**BrF_4_-COOH11**	2.5975(18)	175(2)	2.7921(14)	18	177.45(7)
**IF_4_-OX13**	2.746(3)	173(3)	2.9972(18)	15	173.15(6)
**IF_4_-OX11**	2.690(2)	174(3)	2.8395(18)	20	175.29(7)
**IF_4_-COOH13**	2.550(7)	162	3.093(6)	12	174.8(2)
**IF_4_-COOH12**	2.5285(17)	176(2)	2.7935(14)	21	177.65(5)
**Br-COOH11**	2.626(2)	174(3)			
**Br-COOH12**	2.628(4)	168.4			
**BrF_4_-OX14**	2.693(2)	173(3)			
**BrF_4_-OH12**	2.633(3)	164(5)			
**BrF_4_-OH11**	2.556(3)	158(3)			
**IF_4_-OH16**	2.644(2)	156(3)	2.9218(15)	17	172.05(6)
**IF_4_-OH2**	2.619(2)	158(3)	3.0486(18)	14	174.76(6)
**IF_4_-COOH16**	2.6469(18)	155.7	2.8102(11)	20	170.36(4)
**IF_4_-COOH2**	2.653(5)	174.8	2.997(4)	15	176.48(13)
**Br-COOH2**	2.663(3)	160(3)	3.224(4)	5	170.94(9)
**BrF_4_-OH2**	2.608(2)	153.5	3.009(2)	12	173.82(7)
