# Supplementary material for: A systematic structural study of halogen bonding versus hydrogen bonding within competitive supramolecular systems
Source: IUCrJ. 2015 Jul 30;2(Pt 5):498–510. doi: 10.1107/S2052252515010854 (PMC4547818; doi:10.1107/S2052252515010854)
Supplement: Supplementary file 2 [file m-02-00498-sup2.pdf]

# IUCrJ

**Volume 2 (2015)**


**Supporting information for article:**

## A systematic structural study of halogen bonding *versus* hydrogen bonding within competitive supramolecular systems

**Christer B. Aakeröy, Christine L. Spartz, Sean Dembowski, Savannah Dwyre and John Desper**

## S1. Electrostatic Potential Calculations for Acceptor Molecules

The theoretical electrostatic potential calculations for all of the acceptor molecules were performed using Density Functional Theory (DFT) at the 6-31++G** basis set. The results for all twenty acceptor molecules are shown in the table below.

**Table S1** Electrostatic Potential Calculations for Acceptor Molecules

| Acceptor | Structure | Electrostatic Potential (kJ/mol) | |
|---|---|---|---|
| **1** | 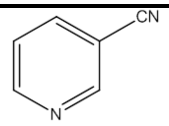 | py = -146.0 | CN = -172.3 |
| **2** | 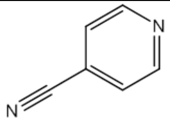 | py = -147.6 | CN = -164.0 |
| **3** | 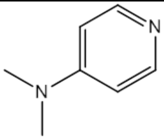 | py = -217.4 | |
| **4** | 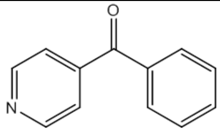 | py = -177.8 | |
| **5** | 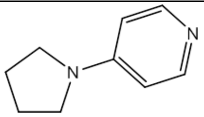 | py = -228.4 | |
| **6** | 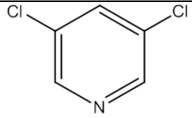 | py = -148.3 | |
| **7** | 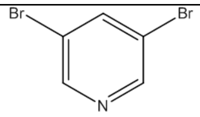 | py = -148.0 | |
| **8** | 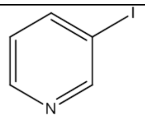 | py = -168.1 | |
| **9** | 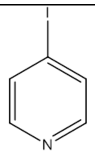 | py = -172.8 | |

| | | | |
|---|---|---|---|
| **10** | 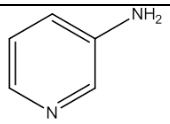 | py = -206.4 | |
| **11** | 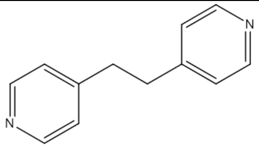 | py = -188.0 | |
| **12** | 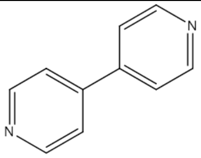 | py = -176.8 | |
| **13** | 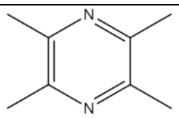 | py = -163.3 | |
| **14** | 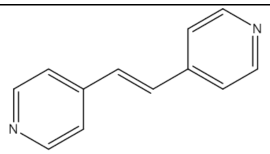 | py = -182.7 | |
| **15** | 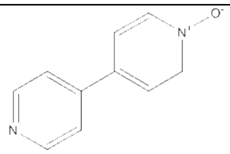 | N-O = -184.0 | py = -174.6 |
| **16** | 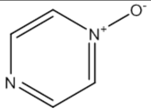 | N-O = -164.9 | py = -148.1 |
| **17** | 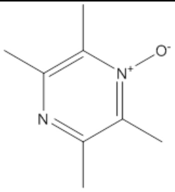 | N-O = -178.4 | py = -155.8 |
| **18** | 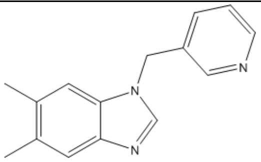 | im = -214.3 | py = -179.6 |
| **19** | 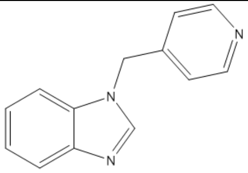 | im = -198.6 | py = -177.9 |

| | | | |
|---|---|---|---|
| **20** | 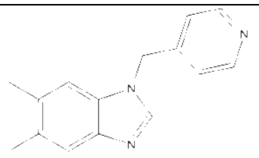 | im = -206.6 | py = -181.4 |

### S2. Melting Point Information

Melting point data was recorded on a Gallenkamp melting point apparatus, and results were non-corrected.

**Table S2** Melting point (MP) data for co-crystals and their donors and acceptors

| D--A | Donor MP (°C) | Acceptor MP (°C) | Co-crystal MP (°C) | Co-crystal MP Between/Outside |
|---|---|---|---|---|
| **$IF_4$-COOH—2** | 151 | 70 | 125-129 | between |
| **$IF_4$-COOH—4** | 151 | 64 | 102-104 | between |
| **$IF_4$-COOH—12** | 151 | 102 | dec. 165-170 | above |
| **$IF_4$-COOH—13** | 151 | 77 | dec. 138-141 | between |
| **$IF_4$-COOH—16** | 151 | 110 | 111-115 | between |
| **$BrF_4$-COOH—11** | 142 | 98 | 137-139 | between |
| **I-COOH—11** | 215 | 98 | 190-192 | between |
| **I-COOH—12** | 215 | 102 | 179-182 | between |
| **Br-COOH—2** | 234 | 70 | 210-221 | between |
| **Br-COOH—3** | 234 | 105 | 151-153 | between |
| **Br-COOH—5** | 234 | 38 | 159-162 | between |
| **Br-COOH—12** | 234 | 102 | dec. 159-164 | between |
| **Br-COOH—11** | 234 | 98 | 140-142 | between |
| **$IF_4$-OX—3** | 174 | 105 | dec. 90-94 | below |
| **$IF_4$-OX—11** | 174 | 98 | 135-142 | between |
| **$IF_4$-OX—13** | 174 | 77 | 133-135 | between |
| **$BrF_4$-OX—14** | 177 | 152 | 141-145 | below |
| **Br-OX—5** | 96 | 38 | 108-112 | above |
| **$IF_4$-OH—2** | 44 | 70 | dec. 97-98 | above |
| **$IF_4$-OH—16** | 44 | 110 | dec. 102-106 | between |
| **$BrF_4$-OH—2** | 40 | 70 | dec. 100-103 | above |
| **$BrF_4$-OH—11** | 40 | 98 | 125-130 | above |
| **$BrF_4$-OH—12** | 40 | 102 | 118-119 | above |

| | | | | |
|---|---|---|---|---|
| **$BrF_4$-OH—13** | 40 | 77 | dec. 119-121 | above |

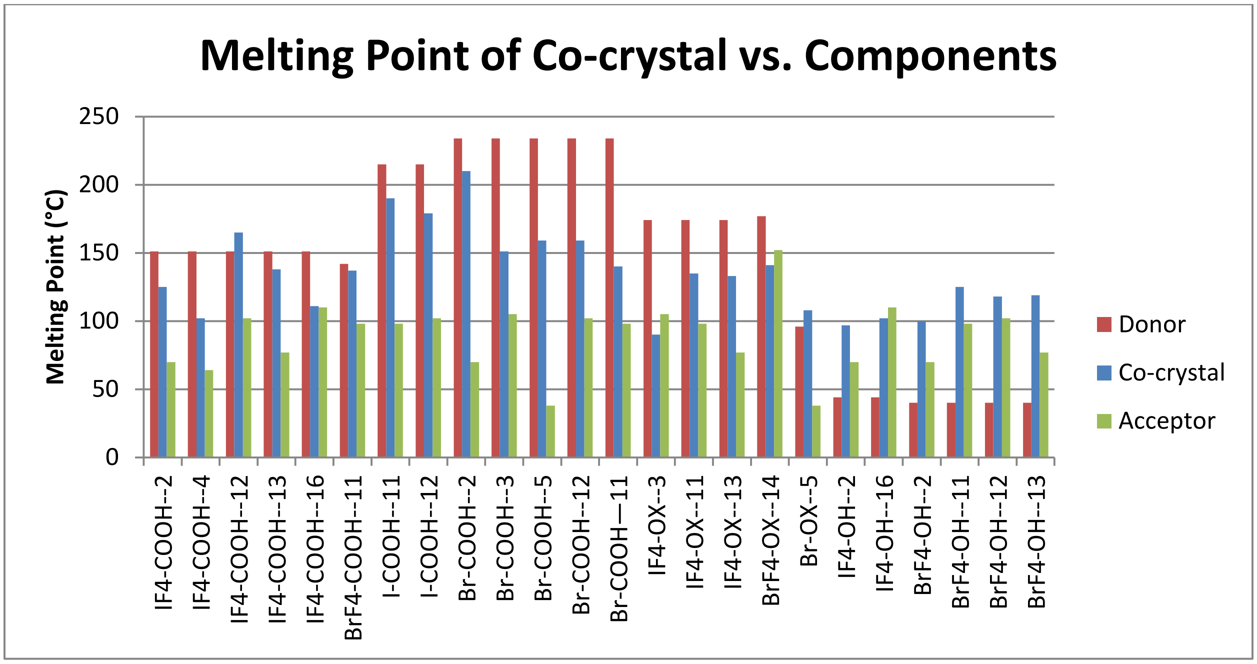


**Figure S1** Melting point data shown graphically. It is noticeable that the melting point of most co-crystals falls between its donor and acceptor's melting points, but the melting point of the phenolic-ligand co-crystal are significantly higher than their components.

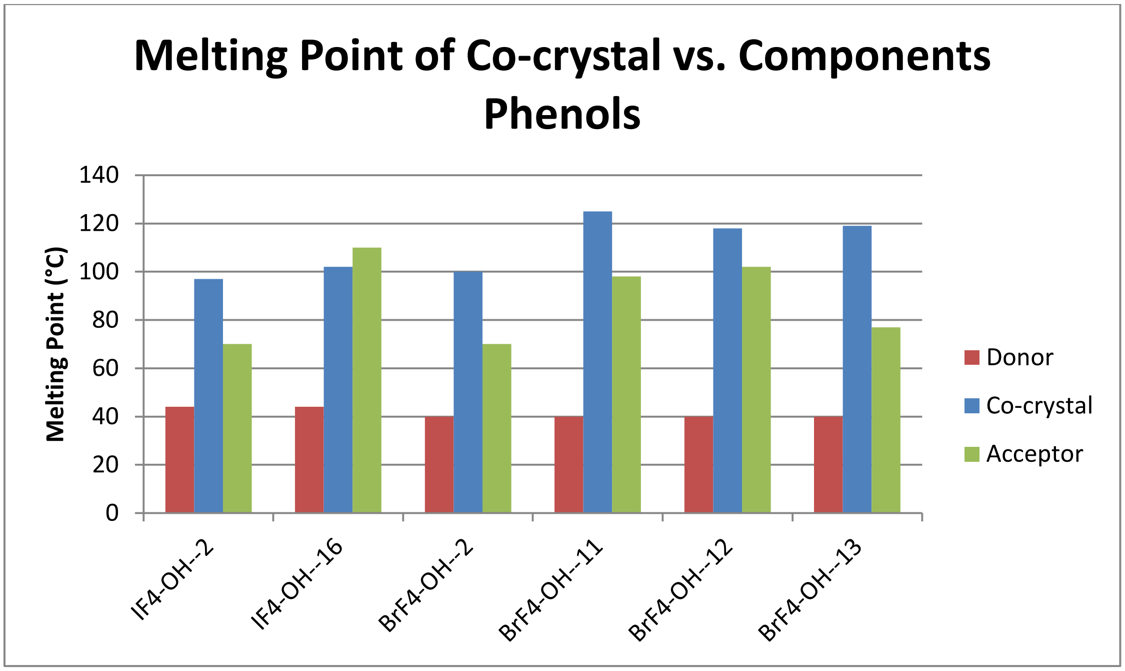


**Figure S2** Melting point data for phenolic-donor ligand co-crystals.

## S3. Crystallographic Information

Samples were analyzed with either a Bruker Kappa APEX II system using CuKα radiation (**$BrF_4$-OX**--14, **$BrF_4$-OH**--11) or a Bruker APEX II system using MoKα radiation (all others). Data collection was carried out using APEX2 software.[i] Initial cell constants were found by small widely separated “matrix” runs. Data collection strategies were determined using COSMO.[ii] Scan speed and scan widths were chosen based on scattering power and peak rocking curves. All datasets were collected at -153°C using an Oxford Croystream low-temperature device.

Unit cell constants and orientation matrix were improved by least-squares refinement of reflections thresholded from the entire dataset. Integration was performed with SAINT,[iii] using this improved unit cell as a starting point. Precise unit cell constants were calculated in SAINT from the final merged dataset. Lorenz and polarization corrections were applied. Multi-scan absorption corrections were performed with TWINABS[iv] for the two non-merohedral twins in this set (**I-COOH**--11and **Br-COOH**--12), and SADABS[v] for the remainder.

Data were reduced with SHELXTL.[vi] The structures were solved in all cases by direct methods without incident. Except as noted below, hydrogen atoms were located in idealized positions and were treated with a riding model. All non-hydrogen atoms were assigned anisotropic thermal parameters. Refinements continued to convergence, using the recommended weighting schemes.

**$IF_4$-OX**--3 The asymmetric unit contains two oxime / amine pairs, which were grouped into two RESIdues.

**Br-OX**--5 The asymmetric unit contains a single oxime / amine pair. The pyrrolidine moiety was disordered at the ethylene bridge, with the population of the two PARTs being set by a free variable. Thermal parameters of the disordered fragment were pairwise constrained with EADP commands. Coordinates of the oxime hydrogen H17 were allowed to refine.

**Br-COOH**--5 The asymmetric unit contains two carboxylic acids and a single amine. The pyrrolidine moiety was disordered at the ethylene bridge, with the population of the two PARTs being set by a free variable. DFIX commands were used to idealize the geometry of the disordered ethylene bridge, and thermal parameters of the disordered fragment were pairwise constrained with EADP commands. Proton transfer from a carboxylic acid to the amine was clearly observed. Coordinates for the remaining carboxylic acid proton and the ammonium proton were allowed to refine.

**Br-COOH**--3 The asymmetric unit contains a single carboxylic acid / amine pair. Proton transfer from carboxylic acid to amine was clearly observed. Coordinates of the ammonium proton were allowed to refine.

**$IF_4$-COOH**--4 The asymmetric unit contains a single carboxylic acid and two amines. The iodo acid was disordered in a head-to-tail fashion, with the population of the two PARTs being set by a free variable. A

SAME command was used to restrain the geometries of the two independent iodo acids. The structure is nearly centrosymmetric, with the amines and the two acid PARTs nearly related by inversion centers. However, the ratio of iodo acids was significantly different from 50:50, with the final refined FVAR = 0.464(1). Only the iodo atoms could be refined anisotropically. Attempts to assign anisotropic thermal variables to the remaining atoms were unsuccessful: the refinement became unstable and the fit did not improve. The data were handled as a eacemic twin.

**I-COOH--**12 The asymmetric unit contains a single carboxylic acid / amine pair. The iodo acid and diamine were both disordered in a head-to-tail fashion. A SAME command was used to restrain the geometries of the two halves of the diamine. Thermal parameters for the two halves of the disordered diamine were pairwise constrained with EADP commands. The ratio of both species was eventually constrained to 50:50 since neither refined to a value significantly different from this ratio.

**I-COOH--**11 The crystal was a non-merohedral twin, and the data were processed with TWINABS. The asymmetric unit contains a single iodo acid / diamine pair. The iodo acid was disordered in a head-to-tail fashion, with the population of the two PARTs being set by a free variable. A SAME command was used to restrain the geometries of the two independent iodo acids. The structure is nearly centrosymmetric, with the diamine and the two diacid PARTs nearly related by inversion centers. However, the ratio of iodo acids was significantly different from 50:50, with the final refined FVAR = 0.623(4). Thermal parameters for both the diamine and iodo acid were pairwise constrained with EADP commands.

**$BrF_4$-OH--**13 The asymmetric unit contains a single phenol / amine pair. Coordinates for the phenol hydrogen were allowed to refine.

**$BrF_4$-COOH--**11 The asymmetric unit contains a single bromo acid / diamine pair. The bromo acid was disordered in a head-to-tail fashion, with the population of the two PARTs being set by a free variable. A SAME command was used to restrain the geometries of the two independent bromo acids Both the diamine and the bromo acid pair sit on pseudo inversion centers at ( 1 ¼ ¼ ) and ( -½ ¼ ¾ ) respectively. However, the ratio of bromo acids was substantially different from 50:50, and significantly different from 100:0, with the final refined FVAR = 0.9464(7). Thermal parameters of the disordered bromo acid were constrained pairwise with EADP commands between atoms in close proximity. Proton transfer from carboxylic acid to amine was clearly observed. Coordinates for the ammonium proton were allowed to refine.

**$IF_4$-OX--**13 The asymmetric unit contains a single iodo oxime / diamine pair. Coordinates for the oxime hydrogen were allowed to refine.

**$IF_4$-OX--**11 The asymmetric unit contains a single oxime / diamine pair. Coordinates for the oxime hydrogen were allowed to refine.

**$IF_4$-COOH--**13 The asymmetric unit contains a single iodo acid / diamine pair.

**$IF_4$-COOH--**12 The asymmetric unit contains a single iodo acid / diamine pair. Proton transfer from carboxylic acid to amine was clearly observed. Coordinates for the ammonium hydrogen were allowed to refine.

**Br-COOH--**14 The asymmetric unit contains a single bromo acid and a half-diamine. The diamine straddles an inversion center. Coordinates for the carboxylic acid hydrogen were allowed to refine.

**Br-COOH--**12 The crystal was a non-merohedral twin, and the data were processed with TWINABS. The asymmetric unit contains three bromo acid / half-diamine pairs, which were grouped into three RESIdues. One of the three unique half-diamines is related to a symmetry equivalent by an inversion center; the other diamine sits on a general position.

**$BrF_4$-OX--**14 The asymmetric unit contains two oxime / half-diamine pairs. Both unique diamines straddle crystallographic inversion centers. Coordinates for the two oxime hydrogens were allowed to refine.

**$BrF_4$-OH--**12 The asymmetric unit contains a single phenol / half-diamine pair. The diamine straddles a crystallographic inversion center. Coordinates for the phenol hydrogen were allowed to refine.

**$BrF_4$-OH--**11 The asymmetric unit contains a single phenol / half-diamine pair. The diamine straddles a crystallographic inversion center. Coordinates for the phenol hydrogen were allowed to refine.

**$IF_4$-OH--**16 The asymmetric unit contains a single phenol / amine pair. Coordinates for the phenol hydrogen were allowed to refine.

**$IF_4$-OH--**2 The asymmetric unit contains a single phenol / amine pair. Coordinates for the phenol hydrogen were allowed to refine.

**$IF_4$-COOH--**16 The asymmetric unit contains a single iodo acid / amine pair. The iodo acid was disordered in a head-to-tail fashion, with the population of the two PARTs being set by a free variable. A SAME command was used to restrain the geometries of the two independent iodo acids. Thermal parameters of the disordered iodo acid were constrained pairwise with EADP commands between atoms in close proximity.

**$IF_4$-COOH--**2 The asymmetric unit contains two iodo acid / amine pairs. Both unique iodo acids were disordered in a head-to-tail fashion, with the population of the two PARTs being set by free variables. A SAME command was used to restrain the geometries of the four independent iodo acids. Thermal parameters of both disordered iodo acids were constrained pairwise with EADP commands between atoms in close proximity.

**Br-COOH--**2 The asymmetric unit contains a single bromo acid / amine pair. Coordinates for the carboxylic acid hydrogen were allowed to refine.

**$BrF_4$-OH--**2 The asymmetric unit contains a single phenol / amine pair.

## **Table S3** Crystallographic data

| | **$IF_4$-OX--3** | **Br-OX--5** | **Br-COOH--5** | **Br-COOH--3** | **$IF_4$-COOH--4** |
|---|---|---|---|---|---|
| Systematic Name | 4-I-F4-PhCHO oxime, 4-(Me2N)-pyridine | 4-Br-PhCH(=NOH), 4-(1-pyrrolidinyl)-pyridine | 4-(1-pyrrolidinyl)-pyridine, (4-Br-PhCOOH)2 | 4-(Me2N)-pyridine, 4-Br-PhCOOH | 4-I-F4-PhCOOH, 4-benzoylpyridine |
| Formula moiety | $(C_7H_2F_4INO)$ $(C_7H_{10}N_2)$ | $(C_7H_6BrNO)$ $(C_9H_{12}N_2)$ | $(C_9H_{12}N_2)$ $(C_7H_5BrO_2)_2$ | $(C_7H_{10}N_2)$ $(C_7H_5BrO_2)$ | $(C_7HF_4IO_2)$ $(C_{12}H_9NO)_2$ |
| Empirical formula | $C_{14}H_{12}F_4IN_3O$ | $C_{16}H_{18}BrN_3O$ | $C_{23}H_{22}Br_2N_2O_4$ | $C_{14}H_{15}BrN_2O_2$ | $C_{31}H_{19}F_4IN_2O_4$ |
| Molecular weight | 441.17 | 348.24 | 550.24 | 323.19 | 686.38 |
| Color, Habit | colourless plate | colourless prism | colourless prism | colourless prism | colourless prism |
| Crystal system | | | | | |
| Crystal system | monoclinic | triclinic | triclinic | monoclinic | monoclinic |
| Space group, Z | P 21/c, 8 | P -1, 2 | P -1, 2 | P 21/c, 4 | C 2, 4 |
| a, Å | 13.9735(9) | 7.8090(11) | 9.3718(6) | 13.5603(12) | 25.216(2) |
| b, Å | 17.2451(11) | 9.6156(14) | 9.6497(6) | 8.1700(8) | 6.0746(5) |
| c, Å | 13.6166(8) | 11.4520(18) | 14.1666(9) | 12.2863(12) | 19.0419(16) |
| α, ° | 90 | 101.314(5) | 98.672(3) | 90 | 90 |
| β, ° | 105.489(2) | 94.225(5) | 105.769(2) | 92.157(3) | 107.453(3) |
| γ, ° | 90 | 113.541(4) | 110.884(3) | 90 | 90 |
| Volume, $Å^3$ | 3162.1(3) | 761.9(2) | 1107.38(12) | 1360.2(2) | 2782.5(4) |
| Density, $g/cm^3$ | 1.853 | 1.518 | 1.650 | 1.578 | 1.638 |
| Temperature, °K | 120(2) | 120(2) | 120(2) | 120(2) | 120(2) |
| Crystal size, min x mid x max | 0.080 x 0.220 x 0.380 | 0.080 x 0.220 x 0.300 | 0.120 x 0.180 x 0.320 | 0.180 x 0.220 x 0.320 | 0.220 x 0.280 x 0.340 |
| X-ray wavelength, Å | 0.71073 | 0.71073 | 0.71073 | 0.71073 | 0.71073 |
| μ, $mm^{-1}$ | 2.074 | 2.700 | 3.693 | 3.021 | 1.218 |
| Trans min / max | 0.689 / 0.852 | 0.644 / 0.813 | 0.487 / 0.666 | 0.540 / 0.612 | 0.706 / 0.775 |
| $\theta_{min}$, ° | 2.362 | 2.384 | 1.554 | 2.911 | 1.121 |
| $\theta_{max}$, ° | 32.071 | 31.500 | 31.672 | 33.118 | 32.572 |
| Reflections | | | | | |
| collected | 33827 | 16077 | 26091 | 19711 | 20908 |
| independent | 10414 | 4635 | 6911 | 4543 | 8074 |
| observed | 7873 | 3907 | 4408 | 3653 | 7228 |
| Threshold expression | $I > 2\sigma(I)$ | $I > 2\sigma(I)$ | $I > 2\sigma(I)$ | $I > 2\sigma(I)$ | $I > 2\sigma(I)$ |
| $R_1$ (observed) | 0.0431 | 0.0294 | 0.0576 | 0.0282 | 0.0829 |
| $wR_2$ (all) | 0.1109 | 0.0782 | 0.1279 | 0.0671 | 0.2097 |
| Goodness of fit (all) | 1.001 | 1.106 | 1.051 | 1.049 | 1.215 |
| Δρ max / min | 1.700 / -0.743 | 0.526 / -0.329 | 1.965 / -2.734 | 0.406 / -0.381 | 0.989 / -2.474 |
| 2θ limit | 30.000 | 30.000 | 30.000 | 30.000 | 25.242 |
| Completeness to 2θ limit | 0.985 | 0.961 | 0.966 | 0.987 | 0.993 |

| | **I-COOH**--12 | **I-COOH**--11 | **BrF$_4$-OH**--13 | **BrF$_4$-COOH**--11 | **IF$_4$-OX**--13 | **IF$_4$-OX**--11 | **IF$_4$-COOH**--13 | **IF$_4$-COOH**--12 |
|---|---|---|---|---|---|---|---|---|
| Systematic name | 4-iodobenzoic acid, 4,4'-bipyridyl | 4-iodobenzoic acid, 1,2-bis(4-pyridyl)-ethane | 4-Br-F4-PhOH, 2,3,5,6-Me$_4$-pyrazine | 4-Br-F$_4$-PhCOOH, 1,2-bis(4-pyridyl)ethane | 4-I-F$_4$-PhCH=NOH, Me$_4$-pyrazine | 4-I-F$_4$-PhCH=NOH, 1,2-bis(4-pyridyl)ethan e | 4-I-F$_4$-PhCOOH, 2,3,5,6-Me$_4$-pyrazine | 4-I-F$_4$-PhCOOH, 4,4'-bipyridyl |
| Formula moiety | $(C_7H_5IO_2)$ $(C_{10}H_8N_2)$ | $(C_7H_5IO_2)$ $(C_{12}H_{12}N_2)$ | $(C_6HBrF_4O)$ $(C_8H_{12}N_2)$ | $(C_7HBrF_4O_2)$ $(C_{12}H_{12}N_2)$ | $(C_7H_2F_4INO)$ $(C_8H_{12}N_2)$ | $(C_7H_2F_4INO)$ $(C_{12}H_{12}N_2)$ | $(C_7HF_4IO_2)$ $(C_8H_{12}N_2)$ | $(C_7HIF_4O_2)$ $(C_{10}H_8N_2)$ |
| Empirical formula | $C_{17}H_{13}IN_2O_2$ | $C_{19}H_{17}IN_2O_2$ | $C_{14}H_{13}BrF_4N_2O$ | $C_{19}H_{13}BrF_4N_2O_2$ | $C_{15}H_{14}F_4IN_3O$ | $C_{19}H_{14}F_4IN_3O$ | $C_{15}H_{13}F_4IN_2O_2$ | $C_{17}H_9F_4IN_2O_2$ |
| Molecular weight | 404.19 | 432.24 | 381.17 | 457.22 | 455.19 | 503.23 | 456.17 | 476.16 |
| Color, Habit | colorless plate | colourless block | colourless rod | colourless prism | colourless rod | colourless plate | colourless plate | colourless prism |
| Crystal system | triclinic | monoclinic | triclinic | triclinic | monoclinic | monoclinic | orthorhombic | monoclinic |
| Space group, Z | P-1, 1 | Pn, 2 | P-1, 2 | P-1, 2 | P2$_1$/c, 4 | P2$_1$/n, 4 | Pna2$_1$, 4 | P2$_1$/c, 4 |
| a, Å | 6.2888(5) | 10.7653(5) | 4.4175(4) | 4.8727(3) | 4.1573(2) | 6.2801(9) | 17.5616(10) | 12.1699(6) |
| b, Å | 7.4027(6) | 7.3634(4) | 12.5878(13) | 10.7621(7) | 14.8817(8) | 11.2226(15) | 14.8078(9) | 11.1933(6) |
| c, Å | 8.1348(6) | 11.4066(6) | 13.4274(14) | 16.5950(10) | 26.2084(13) | 26.846(4) | 6.1199(3) | 12.2448(6) |
| α, ° | 84.773(3) | 90 | 80.827(4) | 87.808(2) | 90 | 90 | 90 | 90 |
| β, ° | 82.604(3) | 109.025(2) | 82.672(3) | 85.332(2) | 91.3450(10) | 95.661(4) | 90 | 104.562(2) |
| γ, ° | 83.781(2) | 90 | 87.809(4) | 83.646(2) | 90 | 90 | 90 | 90 |
| Volume, Å$^3$ | 372.20(5) | 854.80(8) | 730.96(13) | 861.66(9) | 1621.01(14) | 1882.8(5) | 1591.47(15) | 1614.42(14) |
| Density, g/cm$^3$ | 1.803 | 1.679 | 1.732 | 1.762 | 1.865 | 1.775 | 1.904 | 1.959 |
| Temperature, °K | 120(2) | 120(2) | 120(2) | 120(2) | 120(2) | 120(2) | 120(2) | 120(2) |
| Crystal size, min x mid x max | 0.060 x 0.220 x 0.320 | 0.160 x 0.220 x 0.260 | 0.040 x 0.080 x 0.240 | 0.160 x 0.160 x 0.280 | 0.060 x 0.140 x 0.360 | 0.140 x 0.280 x 0.340 | 0.100 x 0.200 x 0.380 | 0.080 x 0.140 x 0.240 |
| X-ray wavelength, Å | 0.71073 | 0.71073 | 0.71073 | 0.71073 | 0.71073 | 0.71073 | 0.71073 | 0.71073 |
| μ, mm$^{-1}$ | 2.160 | 1.887 | 2.858 | 2.446 | 2.026 | 1.754 | 2.066 | 2.042 |
| Trans min / max | 0.722 / 0.881 | 0.566 / 0.752 | 0.704 / 0.894 | 0.635 / 0.696 | 0.750 / 0.888 | 0.703 / 0.791 | 0.580 / 0.820 | 0.787 / 0.854 |
| $\theta_{min}$, ° | 2.533 | 2.260 | 2.072 | 1.232 | 2.737 | 1.968 | 1.799 | 1.729 |
| $\theta_{max}$, ° | 33.117 | 32.191 | 31.663 | 32.880 | 33.142 | 32.029 | 32.541 | 32.565 |
| Reflections | | | | | | | | |
| collected | 8430 | 11346 | 11188 | 23701 | 19470 | 28823 | 12591 | 26566 |
| independent | 2379 | 11346 | 4088 | 6066 | 5424 | 6405 | 4502 | 5816 |
| observed | 2260 | 9582 | 3210 | 5268 | 5024 | 5231 | 3349 | 5225 |
| Threshold expression | I > 2σ(I) | I > 2σ(I) | I > 2σ(I) | I > 2σ(I) | I > 2σ(I) | I > 2σ(I) | I > 2σ(I) | I > 2σ(I) |
| R1 (observed) | 0.0231 | 0.0577 | 0.0504 | 0.0332 | 0.0263 | 0.0287 | 0.0507 | 0.0221 |
| $wR_2$ (all) | 0.0563 | 0.1498 | 0.1370 | 0.0820 | 0.0699 | 0.0787 | 0.1539 | 0.0587 |
| Goodness of fit (all) | 1.049 | 1.120 | 1.052 | 1.042 | 1.227 | 1.026 | 1.066 | 1.056 |
| Δρ max / min | 0.464 / -0.275 | 0.589 / -1.177 | 1.089 / -1.721 | 0.676 / -0.676 | 0.997 / -0.920 | 0.769 / -0.564 | 3.160 / -2.338 | 0.892 / -0.518 |
| 2θ limit | 27.500 | 25.242 | 27.500 | 30.000 | 25.000 | 30.000 | 30.000 | 25.242 |
| Completeness to 2θ limit | 0.977 | 0.998 | 0.983 | 0.986 | 0.983 | 0.995 | 0.999 | 1.000 |

| | **Br-COOH**--11 | **Br-COOH**--12 | **BrF$_4$-OX**--14 | **BrF$_4$-OH**--12 | **BrF$_4$-OH**--11 |
|---|---|---|---|---|---|
| Systematic name | 1,2-bis(4-pyridyl)ethane, (4-Br-PhCOOH)$_2$ | 4,4'-bipyridyl, (4-Br-PhCOOH)$_2$ | (4-Br-F$_4$-PhCH=NOH)$_2$ 1,2-bis(4-pyridyl)ethylene | (4-Br-F$_4$-PhOH)$_2$, 4,4-bipyridyl | 4-Br-F4-PhOH, 1,2-bis(4-pyridyl)ethane |
| Formula moiety | $(C_{12}H_{12}N_2)$ $(C_7H_5BrO_2)_2$ | $(C_{10}H_8N_2)$ $(C_7H_5BrO_2)_2$ | $(C_7H_2BrF_4NO)_2$ $(C_{12}H_{10}N_2)$ | $(C_6HBrF_4O)_2$ $(C_{10}H_8N_2)$ | $(C_6HBrF_4O)_2$ $(C_{12}H_{12}N_2)$ |
| Empirical formula | $C_{26}H_{22}Br_2N_2O_4$ | $C_{24}H_{18}Br_2N_2O_4$ | $C_{26}H_{14}Br_2F_8N_4O_2$ | $C_{22}H_{10}Br_2F_8N_2O_2$ | $C_{24}H_{14}Br_2F_8N_2O_2$ |
| Molecular weight | 586.27 | 558.22 | 726.23 | 646.14 | 674.19 |
| Color, Habit | colourless plate | colourless needle | colourless plate | colourless prism | colourless prism |
| Crystal system | triclinic | monoclinic | triclinic | monoclinic | monoclinic |
| Space group, Z | P-1, 1 | P2$_1$/c, 6 | P-1, 2 | P2$_1$/c, 2 | C 2/c, 4 |
| a, Å | 7.1108(4) | 11.0467(6) | 5.8370(3) | 13.6342(15) | 25.9125(16) |
| b, Å | 7.4624(4) | 28.9658(18) | 12.8464(5) | 5.8333(6) | 4.6925(3) |
| c, Å | 11.2299(6) | 10.7956(7) | 18.1146(8) | 13.3118(14) | 20.0909(12) |
| α, ° | 93.434(2) | 90 | 103.537(2) | 90 | 90 |
| β, ° | 94.022(2) | 110.603(2) | 96.861(2) | 90.944(7) | 103.464(3) |
| γ, ° | 103.373(2) | 90 | 93.060(2) | 90 | 90 |
| Volume, Å$^3$ | 576.53(5) | 3233.4(3) | 1306.46(10) | 1058.58(19) | 2375.8(3) |
| Density, g/cm$^3$ | 1.689 | 1.720 | 1.846 | 2.027 | 1.885 |
| Temperature, °K | 120(2) | 120(2) | 200(2) | 120(2) | 120(2) |
| Crystal size, min x mid x max | 0.060 x 0.220 x 0.340 | 0.040 x 0.080 x 0.340 | 0.060 x 0.240 x 0.280 | 0.060 x 0.140 x 0.296 | 0.200 x 0.240 x 0.300 |
| X-ray wavelength, Å | 0.71073 | 0.71073 | 1.54178 | 0.71073 | 0.71073 |
| μ, mm$^{-1}$ | 3.553 | 3.796 | 4.781 | 3.925 | 3.502 |
| Trans min / max | 0.576 / 0.815 | 0.587 / 0.863 | 0.535 / 0.762 | 0.644 / 0.799 | 0.429 / 0.541 |
| $\theta_{min}$, ° | 1.823 | 1.969 | 2.532 | 2.989 | 2.085 |
| $\theta_{max}$, ° | 33.165 | 31.550 | 68.191 | 31.506 | 32.568 |
| Reflections | | | | | |
| collected | 13290 | 17575 | 16525 | 11348 | 11464 |
| independent | 4144 | 17575 | 4377 | 3352 | 4064 |
| observed | 3415 | 9657 | 3936 | 2301 | 3043 |
| Threshold expression | I > 2\s(I) | I > 2\s(I) | I > 2\s(I) | I > 2\s(I) | I > 2\s(I) |
| R1 (observed) | 0.0388 | 0.0603 | 0.0306 | 0.0439 | 0.0384 |
| $wR_2$ (all) | 0.1065 | 0.1604 | 0.1037 | 0.1130 | 0.1085 |
| Goodness of fit (all) | 1.021 | 0.999 | 0.862 | 1.057 | 1.052 |
| Δρ max / min | 0.771 / -1.014 | 1.013 / -1.031 | 0.254 / -0.539 | 0.555 / -1.123 | 0.946 / -0.966 |
| 2θ limit | 32.500 | 30.000 | 66.000 | 30.000 | 27.500 |
| Completeness to 2θ limit | 0.969 | 0.992 | 0.947 | 0.982 | 0.986 |

| | **$IF_4$-OH**--16 | **$IF_4$-OH**--2 | **$IF_4$-COOH**--16 | **$IF_4$-COOH**--2 | **Br-COOH**--2 | **$BrF_4$-OH**--2 |
|---|---|---|---|---|---|---|
| Systematic name | pyrazine 1-oxide, 4-I-F4-PhOH | 4-I-F4-PhOH, isonicotinonitrile | 4-I-F4-PhCOOH, pyrazine 1-oxide | 4-I-F4-PhCOOH, isonicotinonitrile | 4-Br-PhCOOH, isonicotinonitrile | 4-Br-F4-PhOH, isonicotinonitrile |
| Formula moiety | $(C_4H_4N_2O)$ $(C_6HF_4IO)$ | $(C_6HF_4IO)$ $(C_6H_4N_2)$ | $(C_7HF_4IO_2)$ $(C_4H_4N_2O)$ | $(C_7HIF_4O_2)$ $(C_6H_4N_2)$ | $(C_7H_5BrO_2)$ $(C_6H_4N_2)$ | $(C_6HBrF_4O)$ $(C_6H_4N_2)$ |
| Empirical formula | $C_{10}H_5F_4IN_2O_2$ | $C_{12}H_5F_4IN_2O$ | $C_{11}H_5F_4IN_2O_3$ | $C_{13}H_5F_4IN_2O_2$ | $C_{13}H_9BrN_2O_2$ | $C_{12}H_5BrF_4N_2O$ |
| Molecular weight | 388.06 | 396.08 | 416.07 | 424.09 | 305.13 | 349.09 |
| Color, Habit | bronze plate | gold rod | colourless prism | colourless plate | colourless plate | colourless plate |
| Crystal system | monoclinic | monoclinic | triclinic | monoclinic | triclinic | monoclinic |
| Space group, Z | $P2_1/n$, 4 | $P2_1/n$, 4 | P-1, 2 | $P2_1/c$, 8 | P-1, 2 | $P2_1/n$, 4 |
| a, Å | 11.701(2) | 12.7258(15) | 4.2485(3) | 7.4234(3) | 7.3630(12) | 12.2763(19) |
| b, Å | 4.6121(9) | 4.9322(6) | 11.6204(8) | 35.8851(18) | 7.4800(12) | 5.0037(8) |
| c, Å | 21.594(4) | 20.729(2) | 13.8288(10) | 10.0949(5) | 12.446(2) | 20.666(3) |
| α, ° | 90 | 90 | 110.206(2) | 90 | 80.617(8) | 90 |
| β, ° | 103.591(6) | 106.074(3) | 95.9700(10) | 101.028(2) | 84.893(7) | 106.425(4) |
| γ, ° | 90 | 90 | 98.5310(10) | 90 | 62.018(5) | 90 |
| Volume, $Å^3$ | 1132.7(4) | 1250.2(3) | 624.68(8) | 2639.5(2) | 597.19(17) | 1217.7(3) |
| Density, $g/cm^3$ | 2.275 | 2.104 | 2.212 | 2.134 | 1.697 | 1.904 |
| Temperature, °K | 120(2) | 120(2) | 120(2) | 120(2) | 120(2) | 120(2) |
| Crystal size, min x mid x max | 0.080 x 0.260 x 0.360 | 0.060 x 0.120 x 0.320 | 0.200 x 0.260 x 0.320 | 0.060 x 0.240 x 0.280 | 0.060 x 0.240 x 0.440 | 0.080 x 0.140 x 0.340 |
| X-ray wavelength, Å | 0.71073 | 0.71073 | 0.71073 | 0.71073 | 0.71073 | 0.71073 |
| μ, $mm^{-1}$ | 2.881 | 2.607 | 2.626 | 2.483 | 3.436 | 3.423 |
| Trans min / max | 0.660 / 0.802 | 0.645 / 0.859 | 0.544 / 0.622 | 0.719 / 0.865 | 0.532 / 0.820 | 0.566 / 0.771 |
| $\theta_{min}$, ° | 1.825 | 2.045 | 1.984 | 2.132 | 1.658 | 3.079 |
| $\theta_{max}$, ° | 33.718 | 32.027 | 33.138 | 32.575 | 32.575 | 32.560 |
| Reflections | | | | | | |
| collected | 8224 | 15519 | 18348 | 22625 | 12685 | 9843 |
| independent | 3766 | 4266 | 4376 | 8978 | 3871 | 3721 |
| observed | 3281 | 3824 | 4272 | 6685 | 3201 | 3044 |
| Threshold expression | I > 2\s(I) | I > 2\s(I) | I > 2\s(I) | I > 2\s(I) | I > 2\s(I) | I > 2\s(I) |
| R1 (observed) | 0.0238 | 0.0317 | 0.0181 | 0.0471 | 0.0499 | 0.0423 |
| $wR_2$ (all) | 0.0625 | 0.0870 | 0.0472 | 0.1218 | 0.1351 | 0.1149 |
| Goodness of fit (all) | 1.009 | 1.098 | 1.045 | 1.044 | 1.050 | 1.058 |
| Δρ max / min | 0.710 / -0.825 | 1.195 / -1.998 | 0.623 / -0.654 | 1.501 / -1.031 | 1.551 / -1.673 | 1.486 / -1.422 |
| 2θ limit | 30.000 | 25.242 | 30.000 | 30.000 | 30.000 | 30.000 |
| Completeness to 2θ limit | 0.970 | 0.990 | 0.994 | 0.975 | 0.996 | 0.958 |

[i] APEX2 v2013.10-0, © 2013, Bruker Analytical X-ray Systems, Madison, WI.
[ii] COSMO v1.61, © 1999 - 2009, Bruker Analytical X-ray Systems, Madison, WI.
[iii] SAINT v8.34a, © 1997 - 2013, Bruker Analytical X-ray Systems, Madison, WI.
[iv] TWINABS v2012/1, © 2012, Bruker Analytical X-ray Systems, Madison, WI.
[v] SADABS v2012/1, © 2012, Bruker Analytical X-ray Systems, Madison, WI.
[vi] SHELXTL v2013/4, © 2013, Bruker Analytical X-ray Systems, Madison, WI.
